# A phenomics approach for antiviral drug discovery

**DOI:** 10.1186/s12915-021-01086-1

**Published:** 2021-08-02

**Authors:** Jonne Rietdijk, Marianna Tampere, Aleksandra Pettke, Polina Georgiev, Maris Lapins, Ulrika Warpman-Berglund, Ola Spjuth, Marjo-Riitta Puumalainen, Jordi Carreras-Puigvert

**Affiliations:** 1 Department of Pharmaceutical Biosciences and Science for Life Laboratory, Uppsala University, Box 591, SE-751 24 Uppsala, Sweden; 2 Department of Oncology and Pathology and Science for Life Laboratory, Karolinska Institutet, SE-171 76 Stockholm, Sweden; 3 National Veterinary Institute, SE-756 51 Uppsala, Sweden

**Keywords:** Phenomics, Morphological profiling, Cell Painting, Drug discovery, Antiviral

## Abstract

**Background:**

The emergence and continued global spread of the current COVID-19 pandemic has highlighted the need for methods to identify novel or repurposed therapeutic drugs in a fast and effective way. Despite the availability of methods for the discovery of antiviral drugs, the majority tend to focus on the effects of such drugs on a given virus, its constituent proteins, or enzymatic activity, often neglecting the consequences on host cells. This may lead to partial assessment of the efficacy of the tested anti-viral compounds, as potential toxicity impacting the overall physiology of host cells may mask the effects of both viral infection and drug candidates. Here we present a method able to assess the general health of host cells based on morphological profiling, for untargeted phenotypic drug screening against viral infections.

**Results:**

We combine Cell Painting with antibody-based detection of viral infection in a single assay. We designed an image analysis pipeline for segmentation and classification of virus-infected and non-infected cells, followed by extraction of morphological properties. We show that this methodology can successfully capture virus-induced phenotypic signatures of MRC-5 human lung fibroblasts infected with human coronavirus 229E (CoV-229E). Moreover, we demonstrate that our method can be used in phenotypic drug screening using a panel of nine host- and virus-targeting antivirals. Treatment with effective antiviral compounds reversed the morphological profile of the host cells towards a non-infected state.

**Conclusions:**

The phenomics approach presented here, which makes use of a modified Cell Painting protocol by incorporating an anti-virus antibody stain, can be used for the unbiased morphological profiling of virus infection on host cells. The method can identify antiviral reference compounds, as well as novel antivirals, demonstrating its suitability to be implemented as a strategy for antiviral drug repurposing and drug discovery.

**Supplementary Information:**

The online version contains supplementary material available at 10.1186/s12915-021-01086-1.

## Background

In the light of the current coronavirus disease 2019 (COVID-19) pandemic caused by severe acute respiratory syndrome coronavirus 2 (SARS-CoV-2), the development of new therapies against emerging viruses is of high priority. Understanding how viruses affect the host cells is key to identify potential targets for treatment. RNA viruses are characterized by their fast mutation rate resulting in resilience against antiviral drugs, as well as heavy reliance on host pathways for replication [1, 2]. In this context, coronaviruses are an exception to this norm given the existing RNA proofreading machinery within their genome, but nevertheless develop treatment escape mutants [3]. Therefore, host targeting—as well as combination—therapies might be advantageous to battle current and future virus outbreaks. Hence, new methods to profile cellular responses during virus infection that can be used for drug screening are greatly needed.

Image-based morphological profiling of cells, or phenomics, combines high-content imaging with multiparametric analysis of single-cells to study biological and chemical perturbations [4]. The Cell Painting assay is a high-content image-based method for morphological profiling that uses six multiplexed fluorescent dyes imaged in five channels. These dyes reveal eight relevant cellular components that can be used to simultaneously interrogate numerous biological pathways upon a given perturbation [5]. The resulting images can be analysed using traditional image analysis techniques to provide information-rich profiles of individual cells, as well as serve as input for machine or deep learning approaches [6]. This method has been successfully applied to study compound toxicity, predict cell health indicators, detect morphological disease signatures and give insights into the mechanism of action (MoA) of both existing and novel compounds [7–12].

In contrast to target-based screens, where only a limited number of features are quantified to select for a known cellular phenotype, morphological profiling combined with statistical analysis can unravel subtle morphological patterns and provide insights into new pathways or mechanisms [5]. While morphological profiling is gaining ground in the field of pharmacology and toxicology, its employment in the field of virology has been limited [13]. However, with the increased momentum in virology research caused by the spread of the SARS-CoV-2, morphological profiling is progressively being implemented as a strategy for the selection and optimization of drugs, and drug combinations, as potential antiviral therapies [14–17].

Immunofluorescence-based antiviral screening methods commonly rely on the use of specific antibodies for the visualization and quantification of virus-infected cells. Typically, proteins that are expressed throughout the replication cycle of the virus, and reliably represent the infection status, are the proteins of choice to be detected by immunofluorescence. Although different viral proteins can be expressed at varying abundance throughout the replication cycle, viral structural and nucleocapsid proteins are often chosen to detect the infection, for instance the Zika virus envelope protein, the Ebola virus structural VP40 protein and SARS-CoV-2 Nucleocapsid protein, and have been exploited in immunofluorescence-based antiviral drug screens [18–21]. Immunofluorescence-based methods have been proven successful for identifying drugs that directly affect cell survival and virus infection, but lack the depth to provide detailed information about the effect on the host cell [21, 22]. Here we describe a novel phenomics approach, combining morphological profiling with antibody-based detection of virus infection in a single assay using human coronavirus 229E (CoV-229E)-infected MRC-5 primary lung fibroblasts, in the absence or presence of several known and novel antiviral compounds. We developed an automated image analysis pipeline for the identification of infected and non-infected cells, generating hundreds of morphological measurements to study host-cell biology at a single-cell level. We used the resulting morphological profiles to capture a virus-induced phenotypic signature and show how these profiles can be used to screen for antivirals that reverse the cellular phenotype from an “infected” towards a “non-infected” status, and to potentially identify the MoA of novel antiviral compounds.

## Results

### Cell Painting features capture effects of viral infection

**Fig. 1:**
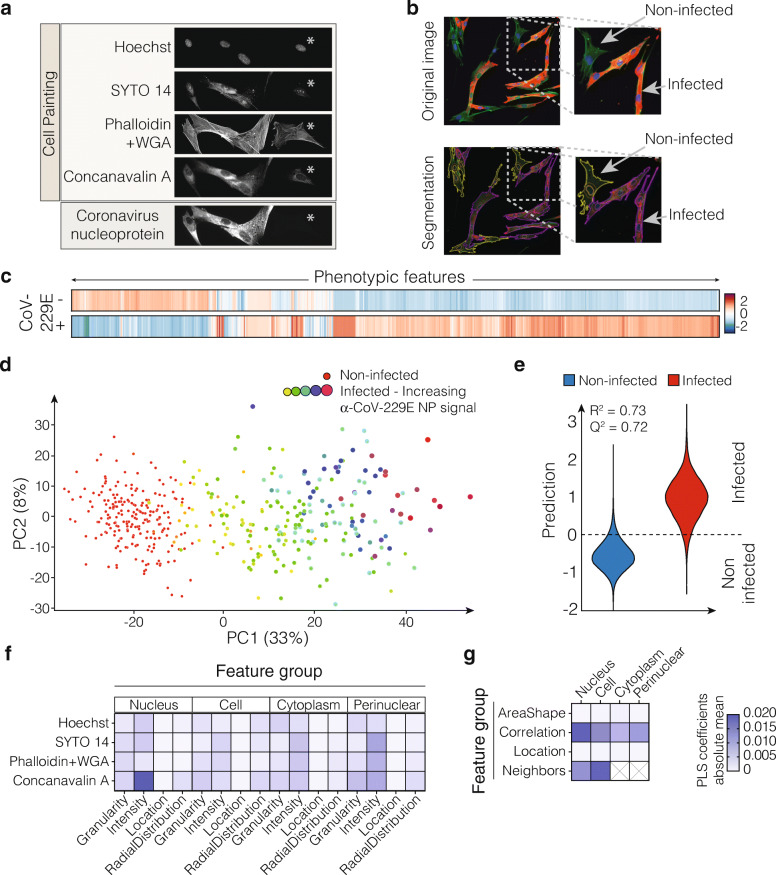
A modified Cell Painting protocol captures a virus-specific morphological signature. **a** MRC-5 lung fibroblast cells infected with Human coronavirus 229E (CoV-229E), stained using Hoechst, SYTO 14, Concanavalin A, Wheat Germ Agglutinin and Phalloidin, in combination with an anti-coronavirus nucleoprotein (NP) antibody. Note the presence of non-infected (asterisk) and infected cells. **b** A representative composite image of infected cells with F-actin in green, nuclei in blue and anti-coronavirus NP antibody in red. Segmentation and classification of individual cells visualized with an outline with infected cells in purple and non-infected cells in yellow. **c** Morphological profiles of non-infected and infected cells (corresponding to the median profiles of both classes). **d** Dimensionality reduction using PCA applied to the extracted CellProfiler features per image, coloured according to their infected or non-infected classification based on NP-specific antibody staining. Percentage of variance explained is indicated by %. **e** With an *R*^2^ = 0.73 and a *Q*^2^ = 0.72, the PLS-DA prediction model could accurately predict viral infection on cell painting features as illustrated by the plot for observed vs predicted values, where observed values correspond to classification by NP-specific antibody. **f**, **g** Overview of the importance of each of the feature classes, grouped by module, cell compartment and stain if applicable. Absolute means of PLS-DA loadings indicate the importance of different feature classes associated with viral infection. Higher PLS coefficients indicate higher importance of a given feature group in order to separate a given condition (in this case, infected cells) from the controls (non-infected cells)

In order to investigate how viral infection affects the host cells, the original morphological profiling protocol known as Cell Painting [5] was modified by replacing the mitochondrial stain, which is typically done using MitoTracker, with a virus antibody-based staining. In the original Cell Painting protocol, MitoTracker Deep Red is applied on live cells as the first step, which can be complex to perform in the required biosafety level laboratory for virus experimentation. MitoTracker has also been reported to affect the morphology of some cell lines when applied as a live stain [23, 24]. Therefore to be able to accommodate an antibody in an optimal fluorescence space, to circumvent costly and time-consuming exhaustive optimizations, and in general to simplify the protocol, we replaced MitoTracker with an anti-virus antibody. Incorporation of an antibody against coronavirus nucleoprotein (NP) allowed for the identification of CoV-229E infection at a single-cell level. In parallel, hundreds of parameters were measured from the infected cells using five of the Cell Painting dyes. Specifically, the dyes consisted of Hoechst (DNA), SYTO 14 (nucleoli and cytoplasmic RNA) and fluorophore-conjugated Phalloidin (F-actin), Concanavalin A (mannose residues of glycoproteins, especially Endoplasmic Reticulum (ER)) and Wheat Germ Agglutinin (sialic acid and N-acetylglucosamine moieties of glycoproteins, especially plasma membrane and Golgi) (Fig. 1a and Table 1).

**Table 1:** Specifications overview for stains and antibodies used in the assay and microscope filters and spectra settings

Label	Excitation spectra (nm)	Emission spectra (nm)	Source	Cat-No	Stock concentration	Concentration used	Target
Hoechst 33342	377/50	447/60	Invitrogen	H3570	10 mg/mL	10 μg/mL	DNA (nucleus)
Pan coronavirus Monoclonal Antibody (FIPV3-70)	-	-	Invitrogen	11500123	1 mg/mL	1:1000	Coronavirus viral nucleoprotein
Goat Anti-Mouse IgG H&L secondary antibody	628/40	692/40	Invitrogen	10739374	2 mg/mL	1:500	
Wheat Germ Agglutinin Alexa Fluor™ 555 Conjugate	562/40	624/40	Invitrogen	W32464	5 mg/mL	15 μg/mL	Golgi, plasma membrane (sialic acid and N-acetylglucosamine moieties of glycoproteins)
Phalloidin Alexa Fluor™ 568 conjugate			Invitrogen	A12380	40x (200U)	10 μL/mL	F-actin
SYTO 14 green	531/40	593/40	Invitrogen	S7576	5 mM	4 μM	Nucleoli, cytoplasmic RNA
Concanavalin A/Alexa Fluor 488 conjugate	482/35	536/35	Invitrogen	C11252	5 mg/mL	80 μg/mL	Endoplasmic reticulum (mannose residues of glycoproteins)

Three phenotypic reference compounds that have been previously reported to produce a distinct morphological phenotype across multiple biologically diverse cell types were used to validate the MRC-5 cell line as a suitable model and to assess reproducibility of the assay [24, 25]. Etoposide and Fenbendazole induced distinct changes in cellular morphology of the MRC-5 human lung fibroblast, characterized by enlarged nucleoli and large multinucleated cells, respectively. In contrast, Metoclopramide, which is known to induce enhanced Golgi staining and fused nucleoli in certain cell types, did not elicit an observable morphological change in this cell line (Additional file 1, Figure S1a).

A CellProfiler image analysis pipeline was designed to distinguish between infected and non-infected cells using the coronavirus NP-specific antibody signal in the perinuclear space as a measure (Fig. 1b). Using this pipeline, a total of 1441 features corresponding to the five fluorescent dyes were extracted at single cell level; subsequently, the cells were labelled according to their infection state based on the NP antibody. After pre-processing, the centred and normalized features were visualized using a heatmap. Clustering of the features highlighted the distinct morphological signature between non-infected and infected cells (Fig. 1c). Principal component analysis (PCA) was used to reduce redundancy and correlation of the features and to facilitate interpretation. PCA analysis revealed a clear separation between infected and non-infected cells on the first principal component, based on the Cell Painting dyes only (Fig. 1d). A gradually increasing separation between non-infected and infected cells coincided with an equally increasing NP antibody signal (Fig. 1d)**.**

To explore what features contributed most to the virus-induced phenotype, we applied partial least-squares discriminant analysis (PLS-DA) on the Cell Painting features using the antibody-based classification results as a label. The PLS-DA model could accurately predict viral infection with a coefficient of determination *R*^2^ = 0.73, and a 3-fold cross-validation resulting in a coefficient of prediction *Q*^2^ = 0.72 (Fig. 1e). Additionally, we calculated the area under the receiver operating characteristics curve (AUROC), which after cross-validation resulted in 0.98 over 1 (Additional file 1, Figure S1b), altogether indicating that the PLS-DA loadings were representative for distinguishing infected from non-infected cells. The PLS-DA loadings were used to indicate important feature classes associated with the CoV-229E-induced phenotype. The virus-induced morphology was characterized by changes in a wide variety of features, distributed over various cell compartments and stains. The most prominent feature groups related to viral infection corresponded to the Concanavalin A and SYTO 14 staining (Fig. 1f, and Additional file 2, Table S1), as well as Correlation and Neighbors features, indicated by higher PLS coefficients in respect to the rest of the feature classes (Fig. 1g, and Additional file 2, Table S1). Overall, our analysis demonstrated the strength of phenomics profiling to identify morphological changes exclusive of CoV-229E-infected cells.

### A novel phenomics approach for the identification of antiviral compounds

**Fig 2:**
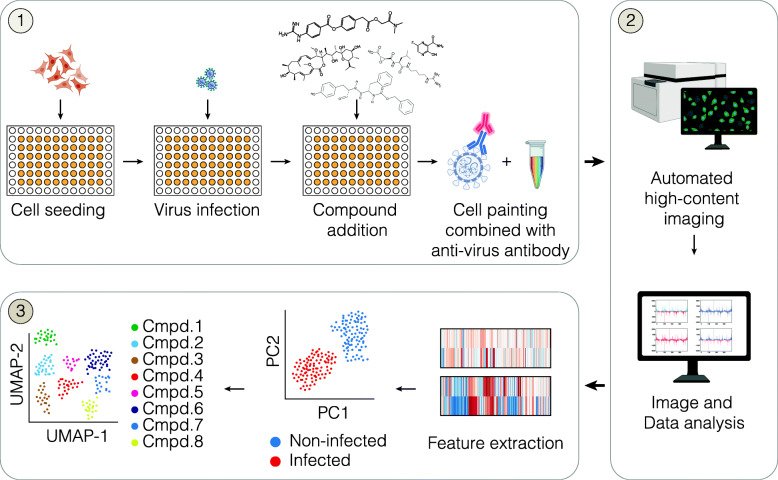
Morphological profiling of virus-infected cells. Overview of the phenomics approach. Cells are seeded in multiwell plates, incubated overnight followed by virus infection. Subsequently, virus-containing media is removed and compounds are added and incubated for 48h followed by fixation. Then, antibody staining and Cell Painting is performed, followed by high content imaging, image analysis and data analysis of the extracted phenotypic features (a more detailed description of the method can be found in Additional file 1 Figure S1c). Illustrations were partially created with BioRender.com

To assess if Cell Painting combined with NP-antibody staining could be leveraged as a tool to profile or screen for antiviral drugs, we designed a phenomics approach including viral infection, compound treatment, cytochemistry protocol, image analysis pipeline and finally data analysis and visualization (Fig. 2, and Additional file 1, Figure S1c). This new method is designed to capture in-depth morphological profiles of the host cells induced by virus infection as well as treatment with potential antiviral compounds.

For the first experimental section (Fig. 2(1)), cells are seeded in multiwell plates and allowed to attach overnight; this is followed by virus infection. In this particular case, MRC5 cells were inoculated with CoV-229E virus for 1 h to maximize viral entry in the host cells [26, 27]. Subsequently, virus-containing media was removed and compounds were added and incubated for 48 h followed by fixation. Post-treatment with antiviral compounds was chosen to mimic a clinical context, in which a patient is treated only after infection is diagnosed. Next, MRC-5 cells were stained using our modified Cell Painting assay including permeabilization and blocking before a coronavirus NP-specific primary antibody was applied. After incubation with the primary antibody, a cocktail of Cell Painting dyes and the secondary antibody was added to the cells.

For the second section of the method (Fig. 2(2)), high-content imaging is used to capture multiple fields of interest covering the wells. A CellProfiler image analysis pipeline is built to extract single-cell features from all the acquired images. The pipeline includes quality control of the images, pre-processing and feature extraction and is universal, requiring only minor adjustments for accurate segmentation and determination of thresholds when applied on different cell lines and viruses. For the third and final section of the method (Fig. 2(3)), a data analysis and data visualization pipeline is built, which more specifically includes data cleansing from corrupted images, normalization and pre-processing of the features, visualization of the data in clustermaps, hierarchical clustering of the morphological profiles and dimensional reduction using PCA and PLS-DA, as well as correlation matrices.

### An antibody-based method identifies Remdesivir and E-64d as potent antiviral compounds

**Fig. 3:**
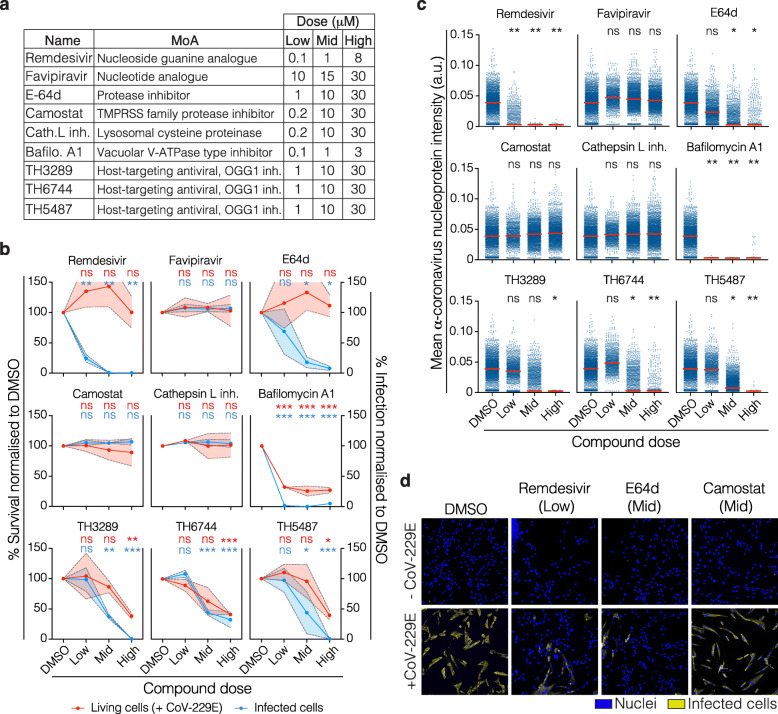
Antiviral efficacy assessment. **a** List of the drugs and compounds with their corresponding mechanism of action (MoA) and doses used in this work. **b** Percentage of survival (red) and percentage of infected cells (blue) normalized to DMSO (infected) control. Coloured intervals indicate the standard deviation of two biological replicates. Two-way ANOVA was performed to assess the statistical significance of each condition (*p<0.05, **p<0.005, ***p<0.0005). **c** Distribution of intensity of the coronavirus anti-NP signal at single-cell level for each tested compound. One-way ANOVA on the means of two biological replicates was performed to assess the statistical significance of each condition (*p<0.05, **p<0.005, ***p<0.0005). **d** Representative images of MRC-5 cells non-infected, or infected with CoV-229E. Hoechst staining for nuclei is visualized in blue, and anti-coronavirus nucleoprotein antibody against CoV-229E in yellow

To compare our phenomics approach to one of the traditional methods for antiviral drugs screening, we first explored the antiviral efficacy of a set of 9 compounds using an NP-specific antibody. The compounds included two direct-acting drugs targeting viral RNA polymerase (Remdesivir and Favipiravir) and seven host-targeting antiviral compounds (E-64d, Camostat, Cathepsin L inhibitor and Bafilomycin A1), including three novel in-house developed broad-spectrum antiviral compounds TH3289, TH6744 and TH5487 (Fig. 3a) [28, 29]. MRC-5 cells were either not infected or infected with CoV-229E and were then exposed to the compounds at three different concentrations (Fig. 3a). We determined cell survival by nuclei count and infection rate by the number of anti-coronavirus NP-positive cells, both in respect to DMSO (Fig. 3b). To avoid systematic errors due to positional effects of the samples, two different randomized layouts for each of the biological replicates were used (Additional file 1, Figure S2a). We assessed interplate variability to ensure stable infection rates between replicates by calculating the Pearson’s correlation coefficients as well as simple linear regression, which on average resulted in Pearson’s *r* > 0.9 and *R*^2^ > 0.8, respectively, indicating high reproducibility (Additional file 1, Figure S2b). In addition, the morphological profiles induced by the phenotypic reference compounds were highly reproducible between biological replicates, with Etoposide and Fenbendazole being particularly distant from the DMSO control, as indicated by PCA analysis (Additional file 1, Figure S2c). Intraplate variability was assessed by calculating the standard deviation (SD) for each replicate condition (Additional file 1, Figure S3a, b).

Antiviral activity with no cellular toxicity was observed upon Remdesivir and E-64d treatment. Treatment with clinically utilized Remdesivir reduced infection by 75% at 0.1 μM and 99% at 1 μM and 8 μM concentrations. At the same time, E-64d treatment resulted in a reduction in viral infection of 31%, 82% or 92% compared to DMSO at 1, 10 or 30 μM concentrations, respectively (Fig. 3b). Neither FDA-approved Favipiravir nor pre-clinical compounds Camostat or Cathepsin L inhibitor reduced the number of infected cells, while Bafilomycin A1 was toxic at all tested concentrations regardless of the presence of the virus (Additional file 1, Figure S4a). Exposure to the broadly active host-targeting antiviral compounds TH3289, TH6744 and TH5487 (Additional file 1, Figure S4b) showed 62%, 56% and 56% reduction in the levels of infected cells, upon 10 μM treatments, respectively (Fig. 3b). The antiviral effect was also confirmed by a decrease in the anti-NP antibody signal (Fig. 3c, d), which was evidently lost for the compounds that displayed toxicity. Finally, the virus-induced cytotoxicity, which resulted in a 20% reduction in the number of cells, was not altered by the use of DMSO as compound vehicle (Additional file 1, Figure S4c).

### Identification of antiviral drugs that reverse infected morphological profiles

**Fig. 4:**
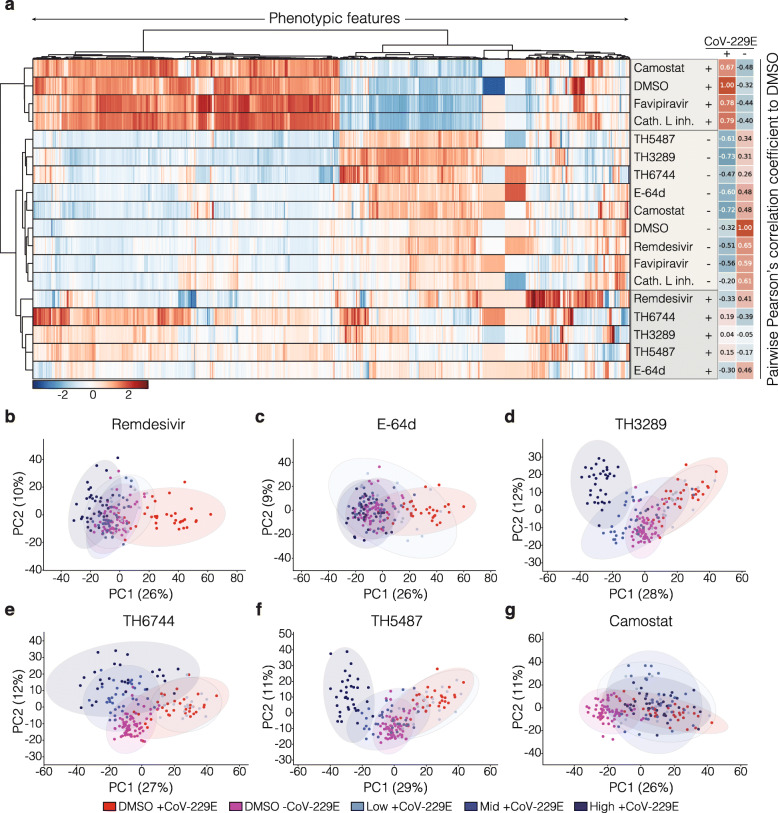
Identification of phenomics profiles of antiviral drugs efficient against coronavirus. **a** Unsupervised hierarchical clustering of morphological profiles of the indicated samples based on their proximity in feature space. Three main groups are shown, infected (+CoV-229E) in the presence of non-active compounds, i.e. Camostat, Favipiravir and Cathepsin L inhibitor, as well as control DMSO (vehicle); non-infected (-CoV-229E) in the presence of all compounds; and +CoV-229E in the presence of the compounds that displayed antiviral effect, i.e. Remdesivir, E-64d, TH3289, TH6477 and TH5487. For this analysis, all compound doses were mean averaged, with the exception of the 30 μM doses for TH3289, TH6744 and TH5487, and all doses of Bafilomycin A1, which resulted in cytotoxicity. Pearson’s correlation coefficients are indicated for each compound and calculated in comparison to DMSO in the absence or presence of CoV-229E. The profiles represent the aggregated mean per compound from two biological replicates and one (for Low dose) or two (for Mid and High doses) technical replicates. **b**–**g** PCA analysis of morphological features upon treatment with Remdesivir, E-64d, TH3289, TH6477, TH5487 and Camostat in non-infected (-CoV-229E) and infected (+CoV-229E) conditions compared to DMSO control. Each dot in the PCA represents one image by taking the mean of all objects in the image. Each data point corresponds to the aggregated image mean from one (for Low dose) or two (for Mid and High doses) technical replicates, and two biological replicates. Percentage of variance explained is indicated by %

In order to demonstrate the use of our approach for drug screening, we assessed the antiviral activity of the aforementioned compounds solely by analysing the morphological profiles of the host cells, in the absence of the NP-antibody. We used unsupervised hierarchical clustering to map similarities between the morphological profiles according to their proximity in feature space averaging all conditions excluding the highest concentrations of TH3289, TH6744 and TH5487, as well as Bafilomycin A1, which were cytotoxic. Hierarchical clustering using the Euclidean metric resulted in three main clusters (Fig. 4a). The first cluster included infected cells treated with non-effective antivirals (Favipiravir, Camostat and Cathepsin L inhibitor). The second cluster contained non-infected conditions (control- and compound- treated). Finally, the third cluster, which was hierarchically linked to the non-infected conditions, included infected cells treated with potent antiviral compounds Remdesivir, E-64d and the in-house developed TH3289, TH6744 and TH5487, suggesting that these compounds rescued or reversed the CoV-229E-induced phenotypic profile (Fig. 4a). The morphological profiles for each of the non-toxic tested compound concentrations are shown in Additional file 1, Figure S5. In order to better visualize the clusterings, we calculated the pairwise Pearson’s correlation coefficients among all conditions, which resulted in a positive correlation between infected cells treated with Remdesivir and E-64d, with control-treated cells in the absence of CoV-229E, confirming the antiviral activity of these compounds (Fig. 4a, Additional file 1, Figures 6a and 7). The morphological features also provided information on the effect of the compound on the cells, as illustrated by the morphological profiles in the absence of virus (Additional file 1, Figure S5). The morphological profiles of TH3289, TH6744 and TH5487, which are structural analogues, clustered closely together as indicated by the dendrogram (Fig. 4a). Similarly, the TMPRSS family protease inhibitor Camostat clustered together with the protease inhibitor E-64d in the absence of the virus (Fig. 4a).

We calculated the Euclidean distances between the non-toxic concentrations of the compounds and the DMSO control in the absence and presence of the virus, which allowed us to rank the compounds by their activity (Additional file 1, Figure S6b). The higher the distance value from the compounds to the DMSO in the presence of CoV-229E, and the lower the distance to non-infected controls, the higher their activity, which confirmed the antiviral effect of Remdesivir and E-64d at all tested concentrations, as well as for TH3289, TH6744 and TH5487 at 10 μM (Additional file 1, Figure S6b).

PCA was used to study the effects of every single compound and dose in more detail. In accordance with the hierarchical clustering, Remdesivir- and E-64d-treated cells grouped together with non-infected control conditions at all tested doses (Fig. 4b, c). Compounds, TH3289, TH6744 and TH5487 showed a dose-dependent response, where treatment with the Mid (10 μM) dose showed a trend towards the non-infected cluster, Low doses (1 μM) clustered with the infected cells, and High doses (30 μM) formed separate clusters (Fig. 4d–f)**.** Treatment with the compounds Camostat, Favipiravir and Cathepsin L inhibitor, which did not reduce the number of infected cells, all clustered with the infected cells in PCA space (Fig. 4g and Additional file 1, Figure S8a, b). Bafilomycin A1 treatment, which was toxic to the cells, as well as cytotoxic doses of the TH compounds, formed clusters separate from both infected and noninfected controls (Additional file 1, Figure S8c).

Uniform Manifold Approximation and Projection (UMAP) was performed to obtain an additional visualization of the distribution of the morphological features for each tested condition, with the exception of cytotoxic doses. This resulted in two clusters, each containing either active (Remdesivir, E-64d, TH3289, TH6744 and TH5487) or inactive compounds (DMSO, Camostat, Favipiravir and Cathepsin L inhibitor) (Additional file 1, Figure S8d). Representative images for each of the compounds are shown in Additional file 1, Figure S9.

In summary, these results demonstrate the suitability of our phenomics approach to identify antiviral drugs by reverting virus-induced phenotypic signatures and by providing information on compounds’ effect on the host cells.

## Discussion

Traditional targeted screens for antiviral drugs often focus on known proteins in the replication cycle of the virus, neglecting the effects of both the virus and the compound on the host cell. On the contrary, morphological cell profiling enables an untargeted approach for the characterization of cellular responses of infected cells with a high spatiotemporal resolution, high throughput, and on a single-cell level. Here, we present a method utilizing Cell Painting combined with a virus-specific antibody to profile host cell responses upon virus infection and demonstrate its potential to serve as a platform for finding effective antiviral compounds.

By adapting the Cell Painting protocol, we were able to detect a distinct virus-induced phenotype for MRC-5 cells infected with CoV-229E (Fig. 1c). The induced phenotype was characterized by changes distributed over various cell compartments and stains, of which the most prominent were observed for Concanavalin A and the SYTO 14. The replication cycle of RNA viruses, including coronaviruses, is closely associated with ER and Golgi networks, ensuring close proximity of replication factors, viral protein processing and ultimately viable virus progeny [30]. Given that Concanavalin A specifically binds to mannose-rich residues of glycoproteins, which are predominantly present in the ER, the increased intensity of ER signal detected in CoV-229E-infected cells can be explained by innate sensing of viral proteins by host ER stress pathways or increased viral glycoprotein content [31]. It should be noted that Cell Painting dyes such as Concanavalin A [32] and SYTO 14 are likely to bind to certain viral-induced or viral-derived targets in the host cell, which could in turn contribute to the generated morphological profiles.

Antibody-based screening methods for antivirals have been successfully used in the past to identify potential candidate treatments [33]. We used this method to benchmark our phenomics approach. According to their antiviral activity, Remdesivir and E64d, which have been previously shown to ameliorate coronavirus infections [34, 35], significantly reduced the percentage of infected cells. Similarly, but with less efficacy, the broad-spectrum antiviral compounds TH3289, TH6744 and TH5487 reduced the number of infected cells, albeit with accompanying toxicity. On the other hand, under our tested conditions, we could not reproduce the previously reported antiviral activities from Favipiravir, Camostat, Cathepsin L inhibitor or Bafilomycin A1 [34–38]. These results may be explained by cell-dependent effects; for instance, it has been reported that MRC-5 cells do not express the TMPRSS2 protease, which is the target of Camostat [39], as well as by the timing, or the selected doses of treatment, which could be addressed by optimization of the experimental conditions in future studies.

Despite the fact that antibody-based screening could identify antiviral compounds, it did not provide further information about the host cells beyond viability. Utilizing our phenomics method we identified antiviral compounds that could reverse the virus-induced morphological profile towards a healthy state. In particular, treatment with Remdesivir, E-64d, TH3289, TH6744 and TH5487 induced morphological profiles that were more similar to non-infected than to infected conditions. Interestingly, as indicated by unsupervised hierarchical clustering, the morphological profiles of TH3289, TH6744 and TH5487, clustered together with drugs that possess much more antiviral efficacy (Fig. 4a). Accordingly, the antiviral activity of Remdesivir and E-64d and the three in-house-developed antivirals was reflected by both their Euclidean distance from the infected controls, as well as their clustering close to the control non-infected condition by PCA analysis, which indicates a potential rescue of the virus-induced phenotype. Of note, since TH6744, TH3289 and TH5487 are host-targeting compounds targeting host-pathways, compound-induced effects are to be expected, which in turn add complexity to the interpretation of the morphological profiles. Altogether, this points to the fact that based solely on the results of the antibody-based method, the in-house developed compounds would have likely been discarded as antivirals given their toxicity. However, according to the results of our phenomics approach, which offers a much higher level of information than simple live/dead assays broadly used in the infectious disease research field, these compounds might have a more potent antiviral efficacy than anticipated, which opens up the possibility for their further development.

A substantial advantage of using morphological profiling to screen for antiviral drugs is the in-depth analysis of the host cell status upon infection as well as upon compound treatment. Thus, despite not being able to recapitulate the antiviral activities of all the tested compounds, we have obtained valuable information that can be used to further understand their biological effect. For instance, in line with several previous studies showing that compounds with similar MoA result in similar phenotypic profiles [4, 40, 41], for novel compounds such as the ones we tested, the comparison of their morphological profiles against the ones of a reference set could aid in the identification of their MoA. In fact, the unsupervised clustering of the morphological profiles (Fig. 4a), as well as the Pearson’s correlation coefficients (Additional file 1, Figures S6a and S7), seem to indicate that, in the presence of the virus, TH3289, TH5487 and to a lesser extent TH6744 induce a similar signature to E-64d. TH3289, TH6744 and TH5487 were originally developed as OGG1 inhibitors; however, it has been already shown that inhibition of OGG1 does not play a role in the antiviral activity of these compounds. In addition, TH6744 could be targeting cellular proteostasis and chaperone-mediated mechanisms [29]; thus, despite the need for validation, the similar signature to E-64d of these compounds (Additional file 1, Figure S6a) could suggest that, in addition to their reported targets, these novel antiviral compounds also have protease inhibitory activity. Despite the very limited number of reference compounds used in our study, this highlights the use of our method not only to identify opportunities for drug repurposing but also for the actual discovery of novel antiviral compounds.

A challenge of morphological profiling is the large amount of data generated, which is accentuated when using single-cell measurements. The analysis strategy of such rich data is open to interpretation and might require specific computational and analytical skills. To facilitate the use of our phenomics approach for visualization of the data and identification of effective antiviral compounds, we have made our data analysis strategy openly available. A comprehensive and detailed guide and toolset on how to analyse morphological profiling data have been previously published [6, 11].

Given the increasing implementation of morphological profiling, the biological interpretation of the morphological changes induced by a given perturbation is a subject of active research [4, 11, 42]. Consequently, the results of this study will benefit from a better understanding of the link between phenomics signatures and the underlying biological mechanism. We have shown how Cell Painting can be applied to complement antibody-based screening for antiviral drugs. The low costs of the chemical stains used in Cell Painting, and the possibility to multiplex with antibody-based screens, will provide high-content information with minimal additional costs, as no extra resources are needed in terms of number of assays and experimental time [43]. Interestingly, our results suggest that Cell Painting alone could potentially be used as a phenotypic screening readout to distinguish infected from non-infected cells, and thus be utilized for antiviral screens without the need for an additional virus-specific antibody, which could also have the potential to further reduce screening costs.

In this work we have taken advantage of CoV-229E because of its relatively low pathogenicity, thereby allowing for experimentation in a biosafety laboratory level-2 (BSL2) lab, and because of its extensive use in the understanding of coronavirus pathogenesis, replication cycle and host-virus interactions. However, similarly to our results for CoV-229E infection, Remdesivir and E-64d have also been reported to effectively prevent SARS-CoV-2 infection in cultured cells, which indicates that our method can be used to also identify antiviral drugs against SARS-CoV-2 [44, 45].

## Conclusions

Our novel phenomics method provides an untargeted readout for the study of virus-induced effects and for the screening of antiviral drugs, at the single-cell level and in a single assay. The morphological signatures obtained using our method can be used to identify the potential MoA of novel compounds by comparing them to those of annotated reference drugs, highlighting the use of this method in drug discovery. Morphological profiling provides high adaptability and scalability to study different cell lines and perturbations. Accordingly, we anticipate that our untargeted approach, with only minor adjustments of the image analysis pipeline, will enable other applications using diverse (human-derived) cell lines, as well as different viruses. Overall, we demonstrated that our phenomics approach can be utilized for the unbiased study of virus-induced effects on host cells that can be leveraged for drug repurposing, as well as for the discovery of novel antiviral compounds.

## Methods

### Biosafety

All experiments in the presence of infectious virus were performed under respective biosafety laboratory (BSL) conditions according to Swedish Work and Health Authorities. Experiments using CoV-229E were performed at BSL2 at Karolinska Institutet.

### Cell culture

MRC-5 cells (from ATCC, Manassas, VA, USA) were cultured in Minimum Essential Media supplemented with 10% (v/v) fetal bovine serum (FBS; Thermo Fisher Scientific, Waltham, MA, USA), 50 U/mL penicillin and 50 μg/mL streptomycin (Thermo Fisher Scientific, Waltham, MA, USA). The cells were maintained at 37 °C under 5% CO2 and the cell culture was routinely tested for Mycoplasma using a luminescence-based MycoAlert kit (Lonza).

### Virus production

CoV-229E (VR-740; from ATCC, Manassas, VA, USA) stocks were amplified in Huh7 cells. Virus titers were determined in Huh7 cells by end-point dilution assay combined with high-throughput immunofluorescence imaging of viral protein staining as previously described [29].

### Compounds

Remdesivir and Favipiravir were purchased from Carbosynth. TH6744, TH3289 and TH5487 were in-house produced and recently described [28, 29]. Compounds Camostat (SML0057), E-64d (E8640), Cathepsin L inhibitor (SCP0110) and Bafilomycin A (B1793) were purchased from Sigma-Aldrich. Three phenotypic reference chemicals that are known to produce morphological phenotypes in a variety of cell lines using the Cell Painting assay were included: Etoposide (E1383), Fenbendazole (F5396) and Metoclopramide (M0763) [24, 25]. DMSO (D2438) was used as compound vehicle (all purchased from Sigma Aldrich).

### Virus infection and compound treatments

Antiviral compounds and phenotypic reference chemicals were dissolved in DMSO to 10 mM solutions and dispensed in 96-well source plates at 500✕ of the final concentration using the D300e Digital Dispenser (Tecan, Männedorf, Switzerland) and stored at −20 °C until use. Compound and virus infection conditions were randomized within the plates and multiple plate layouts were used to compensate for systematic effects related to the well position (Additional file 1, Figure S2a). To further avoid plate and edge-effects, the outer wells were excluded from experimentation. Three doses were chosen for each antiviral compound (final concentration; DMSO 0.3% v/v). A total of 2500 MRC-5 cells per well were seeded in black 96 multiwell plates 24 h before infection (Costar 3603, Corning Life Science, Corning, NY, USA) in 100 μL culture medium. The plates were kept at room temperature for 20 min to aid homogeneous spreading in the wells and incubated overnight at 37 °C at 5% CO2 atmosphere. Cells were infected with a MOI 15 in 50 μL culture medium for 1 h and at least four wells per plate were left uninfected as a mock control. Virus-containing medium was removed, replaced with 80 μL culture medium and 20 μL compound-containing medium in at least two technical duplicates, reaching the final compound concentration. Cells were incubated for 48 h. Cells were fixed in 4% paraformaldehyde (Thermo Fisher Scientific, Waltham, MA, USA) in PBS (Gibco, USA) for 20 minutes, washed twice with PBS and stored at 4°C prior to further processing.

### Cell Painting and antibody detection

To profile morphological features of virus-infected cells, an adapted version of the Cell Painting assay was used [5]. Specific fluorescent probes or fluorophore-conjugated small molecules were used for the detection of various cellular components (Table 1). To accommodate virus-specific antibody detection in parallel to Cell Painting, the dye specific to mitochondria (MitoTracker) from the original cell Painting protocol was replaced with a coronavirus-nucleoprotein specific antibody. Compared to the original cell painting assay consisting of six dyes targeting eight components or cell organelles, here five dyes and a virus-specific antibody were used to target virus nucleoprotein in parallel to seven cell organelles and cell components in a single multiplexed staining assay (Table 1). Representative images of each of the stains can be found in Fig. 1a. Pan coronavirus monoclonal antibody (FIPV3-70, Invitrogen) was combined with a secondary antibody using Alexa Fluor 647 fluorophore (10739374, Invitrogen), chosen for its narrow emission and excitation spectra to limit interference with the other channels. To avoid fading of the chemical stains, in our experience, the secondary antibody is best added together with the chemical dyes, but can also be applied sequentially. Concentrations of secondary antibodies need to be adjusted for a good signal-to-noise ratio in the multiplexed assay. In Table 1**,** working concentrations and specifications for the stains and antibodies are presented.

Fixed cells were washed twice (BioTek, 405 LS washer) and permeabilized by incubation with 80 μL of 0.1% Triton X-100 for 20 min at room temperature, followed by two washes with 1X PBS. Then, 80 μL of 0.2% BSA (A8022) in PBS was added and incubated for 30 min. Next, the plates were washed twice with 1X PBS and 50μL primary antibody was added. The primary antibody was incubated for 4 h at room temperature after which the plates were washed three times for 5 min. A staining solution of Hoechst 33342 (Invitrogen, cat.no H3570), SYTO 14 green (Invitrogen, cat.no S7576), Concanavalin A/Alexa Fluor 488 (Invitrogen, cat.no C11252), Wheat Germ Agglutinin/Alexa Fluor 555 (Invitrogen, cat.no W32464) and Phalloidin/Alexa Fluor 568 (Invitrogen, cat.no A12380) was prepared in PBS in addition to Goat anti-Mouse IgG (H+L) Cross-Adsorbed Secondary Antibody, Alexa Fluor 647 (Invitrogen Catalog # A-21235). Fifty microlitres of staining mixture was added to each well and incubated for 30 min, after which the plates were washed a final three times for 5 min. Plates were either imaged immediately or sealed and kept at 4 °C to be imaged later. All washing steps were performed using a Biotek 405 LS washer; dispensing was done using the Biotek Multiflo FX, multimode dispenser. Plates were protected from light as much as possible.

### Imaging

Fluorescent images were acquired using an Image Xpress Micro XLS (Molecular Devices) microscope with a 20× objective using laser-based autofocus. In total, 9 sites per well were captured using 5 fluorescence channels (DAPI, Cy5, TexasRed, Cy3 and FITC) (Table 1). Examples of images from the different channels are shown in Fig. 1a and Additional file 1, Figures S1a and S9.

### Image analysis pipeline

#### Image quality control and preprocessing

Images were processed and analysed with the open-source image analysis software CellProfiler (available at https://cellprofiler.org/), version 4.0.6 [46]. Prior to analysis, a quality control (QC) pipeline was run on the raw images to detect images with artefacts, which may corrupt the data with false values [6]. We computed various measures to represent a variety of artefacts and used statistical analysis to detect outliers. Images deviating more than 5 standard deviations from the median of FocusScore, MaxIntensity, MeanIntensity, PercentMaximal, PowerLogLogSlope and StdIntensity were flagged, inspected and removed if necessary. To remove out-of-focus images, the PercentMaximal score and PowerLogLogSlope was computed and images with PercentMaximal values higher than 0.25, or PowerLogLogSlope values lower than − 2.3 were removed from the dataset. The QC detected 95 images as outliers, as indicated by one or multiple of the control measures. To assess the reproducibility of the assay and detect possible plate and drift effects across multiple plates, the various quality measures were visualized and inspected (Additional file 1, Figure S10**)**.

In addition, we used cell-level quality control based on the Hoechst staining to detect bright artefacts and clumped cells. Bright artefacts in the DAPI channel were identified using the Identify Primary Object module in CellProfiler; artefacts and surrounding pixels were masked to avoid interference with segmentation.

To correct for uneven illumination in the images, the polynomial illumination correction function was calculated for each plate and each image channel across all cycles resulting in one illumination correction image per channel. The illumination functions were then used in the analysis pipelines to correct each image by dividing by the respective illumination correction image.

### Segmentation

Segmentation based on the DAPI channel was performed by applying a Gaussian blur followed by Otsu thresholding to segment the outlines of each nucleus. The cell objects were segmented using Watershed segmentation using minimum cross-entropy of the cytoplasmic RNA stain, using the nuclei as a seed. The cytoplasm cell compartment was defined as the cell object subtracted from the nuclei. Segmentation of the perinuclear cell compartment was accomplished by the expansion of 20 pixels in the concanavalin A channel and subtraction of the nuclei. Minor adjustments of the parameters are needed to be used between different experimental setups and on different cell lines.

### Feature extraction

After identification of the four cell compartments, phenotypic characteristics were measured using the AreaShape, Correlation, Intensity, Granularity, Location, Neighbors and RadialDistribution modules as provided by CellProfiler [46]. A total of 1441 features were extracted from each object which were exported into csv format for downstream analysis.

### Classification

To classify the cells for infection state, we used a virus NP-specific antibody. The classification module by CellProfiler Analyst was used to find highly indicative features associated with infection by manual annotation of infected and noninfected cells. Accordingly, the mean intensity of the antibody signal in the perinuclear cell compartment was selected for classification of MRC-5 cells infected with CoV-229E. The threshold for infection was defined as the mean plus three standard deviations from the virus NP-specific antibody intensity in the blank control. The set-off was determined on a plate-to-plate basis to avoid bias by batch effects and was checked for accuracy by checking for false positives in non-infected wells.

### Antiviral dose-response and cytotoxicity

Nuclei count was used to determine the toxicity induced by compounds alone, as well as compound and virus combined. Averaged nuclei count per condition were normalized to the DMSO controls. To capture relevant cellular phenotypes, cytotoxic compounds resulting in less than 80% survival were removed from the analysis. Of note, given that TH6744 was previously shown to have antiviral activity, and thus to further investigate this novel compound, the Mid (10 μM) concentration was included for downstream analysis, despite the viability levels being at 63%. Antiviral efficacy was calculated as the percentage of virus-positive cells per well and was normalized to DMSO+virus control which was set to 100%.

### Statistics

All experiments were performed in two separate biological replicates, each consisting of duplicate conditions. Where indicated, plots represent mean ± SD of the biological replicates. Statistical analysis was done using GraphPad Prism version 9.0 (GraphPad Software Inc). Two-way-ANOVA was performed for all the datasets that required comparison among multiple data points within a given experimental condition. Dunnet was applied for the correction of multiple comparisons, and the family-wise alpha threshold and confidence level were 0.05 (95% confidence interval).

### Feature analysis

In order to facilitate the broad use of our phenomics approach, an image analysis pipeline is provided with this work, which is tailored to the application here presented, and once executed will provide the necessary input for subsequent analysis. Downstream analysis of the feature data was performed using Python 3. Prior analysis, the data was cleaned from images with artefacts as identified by the QC pipeline described above. Next, the feature values were mean centred and normalized to unit variance using the fit.transform method of StandardScaler of scikit-learn module (version 0.22.1). Normalization was done across all plates. Invariant features, features with extreme variation (>15 standard deviations) and features with missing data were removed. The features relating to the virus antibody were removed prior analysis of virus-induced morphological profiles.

To compare morphological profiles, the features were presented as cluster maps. Feature normalization ensured that each feature (clustermap column) had a mean of 0 and variance of 1. The normalized features were aggregated to a mean profile per compound. Unsupervised hierarchical clustering on the morphological profiles was computed using the Euclidean metric, and the clustering algorithm using Ward’s method and visualized using the seaborn data visualization library (version 0.11.0). Pearson’s correlation coefficients were pairwise calculated using Panda’s python library function with default settings.

Visualization of the high dimensional data was done using the principal component analysis (PCA) on the centred and normalized features and Uniform Manifold Approximation and Projection (UMAP) using sklearn (version 0.22.1). UMAP visualization was done using 30 neighbours. The image-level feature data was mean averaged to simplify visualization.

Partial least-squares discriminant analysis (PLS-DA) was used to model the virus infection and select features associated with viral infection. *Q*^2^ and *R*^2^ values were calculated for the PLS-DA model, which were estimated by three-fold cross-validation. The absolute mean of PLS-DA loadings were calculated and were grouped by Cell Profiler module (AreaShape, Correlation, Intensity, Granularity, Location, Neighbors, RadialDistribution), cell compartment (nuclei, perinuclear space, cytoplasm, cell) and stain (Hoechst, Concanavalin A, SYTO 14, WGA and Phalloidin).

## Supplementary Information

**Additional file 1: Fig. S1**. Representative images of phenotypic reference compounds, calculated AUROC and phenomics approach overview. a Representative images of the phenotypic reference compounds Fenbendazole (Fen), Etoposide (Eto) and Metoclopramide (Met) to which MRC-5 cells were exposed for 48h at 5 μM, and stained with the indicated Cell Painting dyes or fluorophore conjugates. b Calculated area under the receiver operating characteristics curve (AUROC) = 0.98, which indicates that the PLS-DA loadings are representative for distinguishing infected from non-infected cells. c Detailed overview of the phenomics approach here described. **Fig. S2**. Randomized layouts and reproducibility assessment. a Two different randomized layouts were used for each biological replicate. Compounds are indicated by the colour scheme, concentrations are indicated by L (Low), M (Mid) or H (High), absence or presence of the virus is indicated by -/+ CoV-229E. b Correlation calculation between the two replicate/layouts assessed by Pearson correlation, with a 95% confidence interval, as well as simple linear regression (R^2^). c PCA of the morphological profiles induced by the phenotypic reference compounds for both biological replicate 1 (circle) and 2 (triangle). **Fig. S3**. Intraplate variation. a and b. Mean proportion of infected cells per condition and replicate, with calculated standard deviation SD indicated next to each corresponding dose when applicable. The proportion of infected cells in DMSO conditions was 91.4% +/- 6.9 SD for two biological replicates. **Fig. S4.** Survival of cells exposed to antiviral compounds and structures for the in-house synthesized compounds. a Nuclei count was used to assess the survival of MRC-5 cells exposed to the indicated compounds for 48h. Two-way ANOVA was performed to assess the statistical significance of each condition (*p<0.02, **p<0.002, ***p<0.0002). b Chemical structures for the in-house synthesized compounds TH3289, TH5487 and TH6744. c Average nuclei count per image for DMSO or media conditions, in the presence or absence of CoV-229E. The use of DMSO as vehicle for the compounds did not result in alteration of the cytotoxicity effect of the virus, which resulted in 20% cytotoxicity. Data points are mean values ± SD from two biological duplicates and multiple technical replicates. **Fig. S5.** Morphological profiles and Pearson’s correlation coefficients. Individual morphological profiles presented as heatmaps of every tested compound at non-toxic concentrations, accompanied to the Pearson’s correlation coefficients for each condition in respect to DMSO in the presence or absence of CoV-229E. **Fig. S6.** Pairwise Pearson’s correlations and Euclidean distances calculated for each morphological profile. a Pairwise Pearson’s correlations coefficients for the mean averaged morphological profiles of each tested compound at non-toxic concentrations. The profiles of infected cells treated with Remdesivir and E-64d positively correlate with DMSO in the absence of the virus. b Euclidean distances calculated for each morphological profile in respect to DMSO conditions in the presence or absence of CoV-229E. The higher Euclidean distance of Remdesivir, E-64d, TH3289, TH6744 and TH5487, at the indicated concentrations, reflect antiviral activity. **Fig. S7**. Pairwise Pearson’s correlations coefficients for the morphological profiles of each tested compound at non-toxic concentrations. Fig. S8. PCA of the morphological features of each indicated compound. a - c PCA of morphological features upon treatment with Favipiravir, Cathepsin L inhibitor and Bafilomycin A1 in infected (+CoV-229E) conditions compared to DMSO control (-CoV-229E). Each dot in the PCA represents one image by taking the mean of all objects in the image. Percentage of variance explained is indicated by %. d UMAP analysis results in two clusters each containing either active or inactive antiviral compounds. **Fig. S9.** Representative images of the modified Cell Painting assay, including the virus-NP antibody, for the indicated treatments. **Fig. S10**. Quality control measures for all plates in the experiment. Images deviating more than five standard deviations from the median of FocusScore, MaxIntensity, MeanIntensity, PercentMaximal, PowerLogLogSlope and StdIntensity were flagged as outliers and removed from the analysis.

**Additional file 2: Table S1**. PCA loadings of the first Principal component indicating feature importance (Fig. 1d). The top 20 positively correlated, as well as 20 most negatively correlated features including their loadings on the first principal component.

**Additional file 3:.** Data. Numerical data corresponding to Fig. 3b, Fig. 3c, Additional file 1, Fig. S2b, Additional file 1 Fig. S3, Additional file 1 Fig. S4a and Additional file 1 Fig. S4c.

## Data Availability

All data generated or analysed during this study are included in this published article and its complementary information files. Supporting data values corresponding to Fig. 3b, c, Additional file 1 Fig. S2b, Additional file 1 Fig. S3, Additional file 1 Fig. S4a and Additional file 1 Fig. S4c are included in Additional file 3. All image data, the image analysis pipelines (quality control, illumination correction and feature extraction) and extracted features are publicly available at Figshare: Rietdijk, Jonne; Tampere, Marianna; Pettke, Aleksandra; Georgiev, Polina; Lapins, Maris; Warpman-Berglund, Ulrika; et al. (2021): A phenomics approach for antiviral drug discovery - Images, analysis pipelines and feature data. SciLifeLab. Dataset. 10.17044/scilifelab.14188403.v1 Data analysis code is available in Github: https://github.com/pharmbio/antiviral-phenomics
