# Supplementary material for: A phenomics approach for antiviral drug discovery
Source: BMC Biol. 2021 Aug 2;19:156. doi: 10.1186/s12915-021-01086-1 (PMC8325993; doi:10.1186/s12915-021-01086-1)
Supplement: Supplementary file 1 — Additional file 1: Fig. S1. Representative images of phenotypic reference compounds, calculated AUROC and phenomics approach overview. a Representative images of the phenotypic reference compounds Fenbendazole (Fen), Etoposide (Eto) and Metoclopramide (Met) to which MRC-5 cells were exposed for 48h at 5 μM, and stained with the indicated Cell Painting dyes or fluorophore conjugates. b Calculated area under the receiver operating characteristics curve (AUROC) = 0.98, which indicates that the PLS-DA loadings are representative for distinguishing infected from non-infected cells. c Detailed overview of the phenomics approach here described. Fig. S2. Randomized layouts and reproducibility assessment. a Two different randomized layouts were used for each biological replicate. Compounds are indicated by the colour scheme, concentrations are indicated by L (Low), M (Mid) or H (High), absence or presence of the virus is indicated by -/+ CoV-229E. b Correlation calculation between the two replicate/layouts assessed by Pearson correlation, with a 95% confidence interval, as well as simple linear regression (R2). c PCA of the morphological profiles induced by the phenotypic reference compounds for both biological replicate 1 (circle) and 2 (triangle). Fig. S3. Intraplate variation. a and b. Mean proportion of infected cells per condition and replicate, with calculated standard deviation SD indicated next to each corresponding dose when applicable. The proportion of infected cells in DMSO conditions was 91.4% +/- 6.9 SD for two biological replicates. Fig. S4. Survival of cells exposed to antiviral compounds and structures for the in-house synthesized compounds. a Nuclei count was used to assess the survival of MRC-5 cells exposed to the indicated compounds for 48h. Two-way ANOVA was performed to assess the statistical significance of each condition (*p<0.02, **p<0.002, ***p<0.0002). b Chemical structures for the in-house synthesized compounds TH3289, TH5487 and TH6744. c Averag [file 12915_2021_1086_MOESM1_ESM.pdf]

Fig.S1

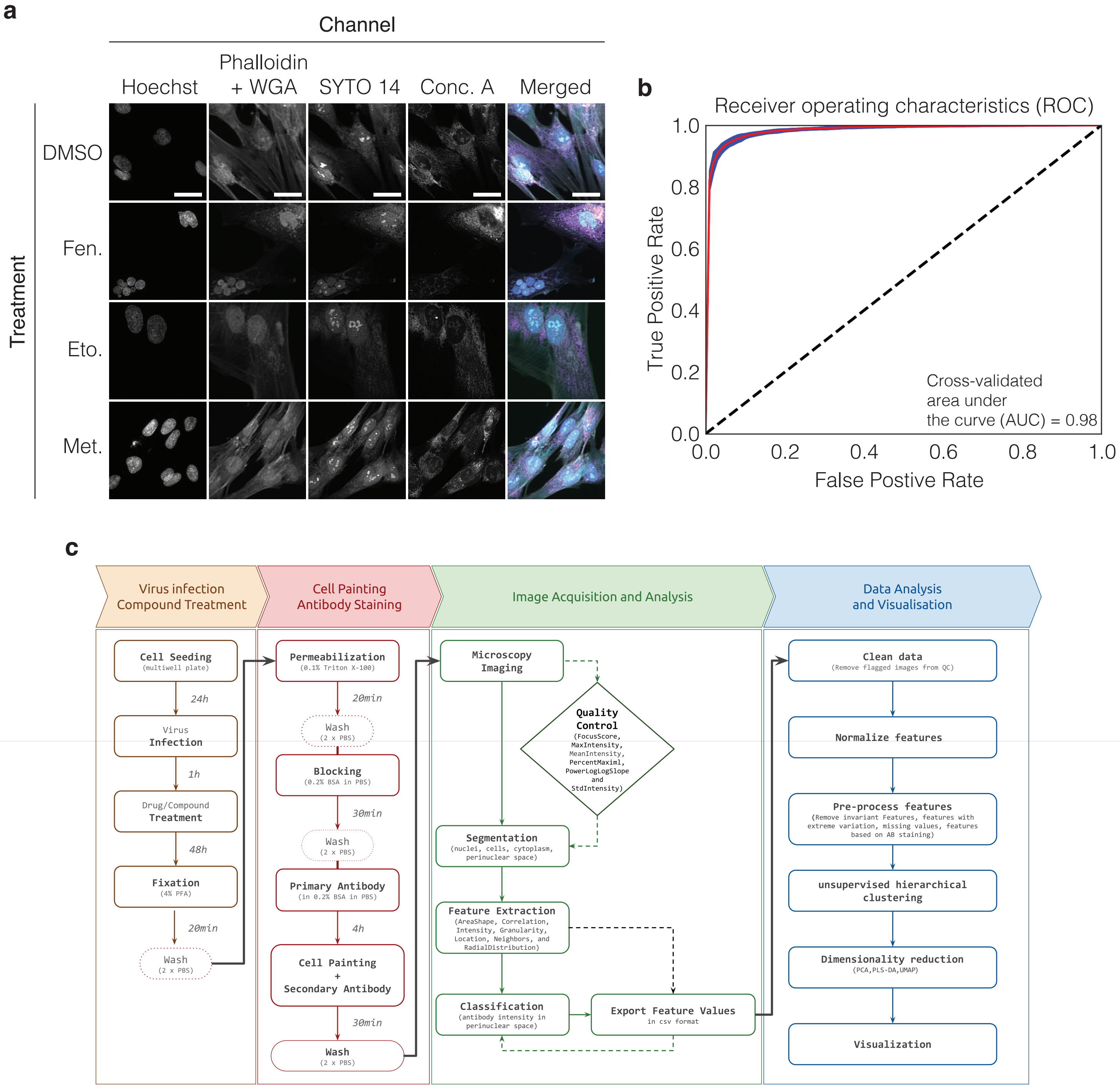
a
Channel
Hoechst
Phalloidin + WGA
SYTO 14
Conc. A
Merged
Treatment
DMSO
Fen.
Eto.
Met.
b
Receiver operating characteristics (ROC)
True Positive Rate
False Postive Rate
0.0
0.2
0.4
0.6
0.8
1.0
Cross-validated area under the curve (AUC) = 0.98
c
Virus infection Compound Treatment
Cell Painting Antibody Staining
Image Acquisition and Analysis
Data Analysis and Visualisation
Cell Seeding (multiwell plate)
24h
Virus Infection
1h
Drug/Compound Treatment
48h
Fixation (4% PFA)
20min
Wash (2 x PBS)
Permeabilization (0.1% Triton X-100)
20min
Wash (2 x PBS)
Blocking (0.2% BSA in PBS)
30min
Wash (2 x PBS)
Primary Antibody (in 0.2% BSA in PBS)
4h
Cell Painting + Secondary Antibody
30min
Wash (2 x PBS)
Microscopy Imaging
Quality Control (FocusScore, MaxIntensity, MeanIntensity, PercentMaximl, PowerLogLogSlope and StdIntensity)
Segmentation (nuclei, cells, cytoplasm, perinuclear space)
Feature Extraction (AreaShape, Correlation, Intensity, Granularity, Location, Neighbors, and RadialDistribution)
Classification (antibody intensity in perinuclear space)
Export Feature Values in csv format
Clean data (Remove flagged images from QC)
Normalize features
Pre-process features (Remove invariant Features, features with extreme variation, missing values, features based on AB staining)
unsupervised hierarchical clustering
Dimensionality reduction (PCA,PLS-DA,UMAP)
Visualization

Fig.S2

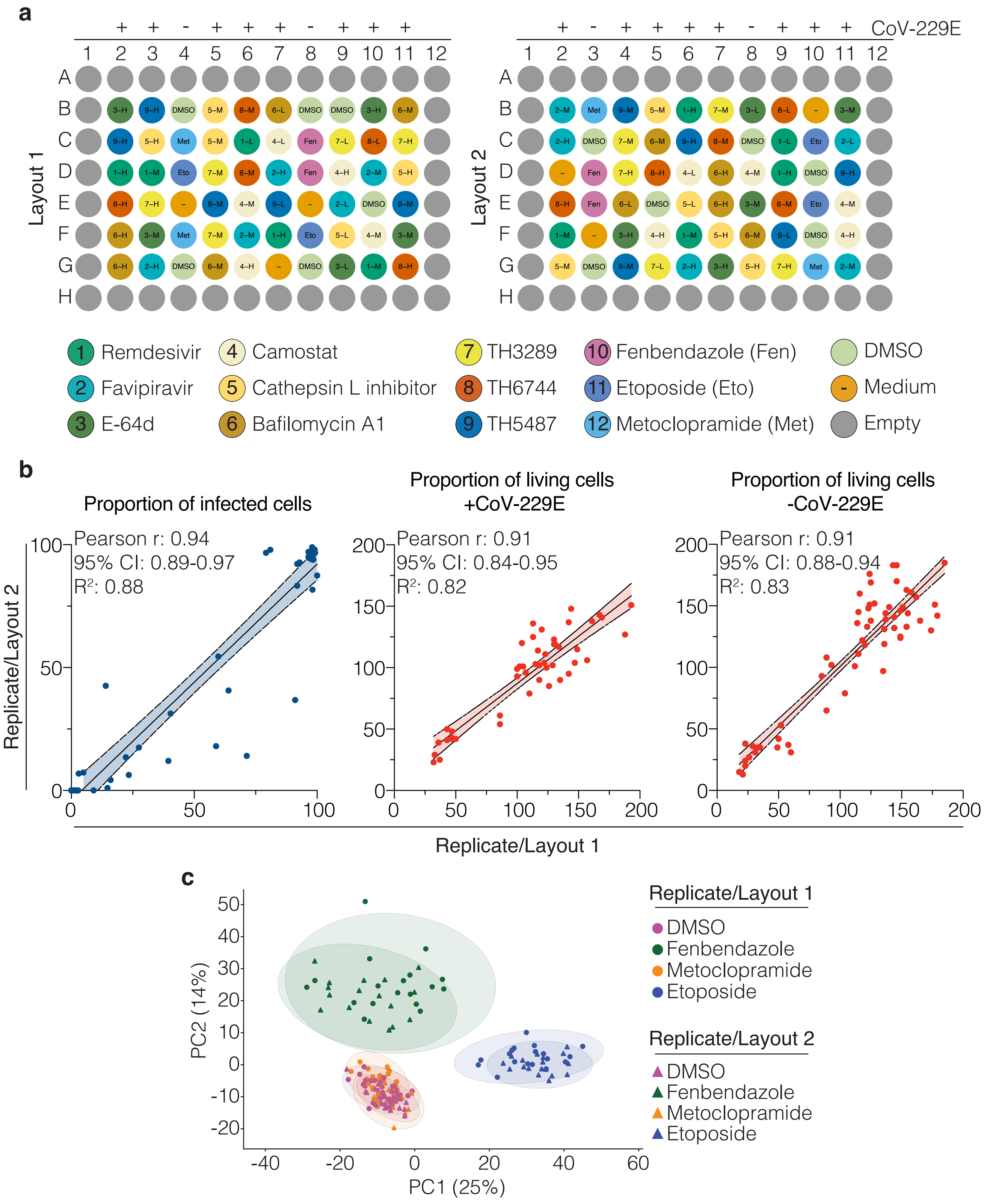

a
CoV-229E
Layout 1
Layout 2
1 Remdesivir
2 Favipiravir
3 E-64d
4 Camostat
5 Cathepsin L inhibitor
6 Bafilomycin A1
7 TH3289
8 TH6744
9 TH5487
10 Fenbendazole (Fen)
11 Etoposide (Eto)
12 Metoclopramide (Met)
DMSO
- Medium
Empty
b
Proportion of infected cells
Pearson r: 0.94
95% CI: 0.89-0.97
R²: 0.88
Proportion of living cells +CoV-229E
Pearson r: 0.91
95% CI: 0.84-0.95
R²: 0.82
Proportion of living cells -CoV-229E
Pearson r: 0.91
95% CI: 0.88-0.94
R²: 0.83
Replicate/Layout 2
Replicate/Layout 1
c
PC2 (14%)
PC1 (25%)
Replicate/Layout 1
DMSO
Fenbendazole
Metoclopramide
Etoposide
Replicate/Layout 2
DMSO
Fenbendazole
Metoclopramide
Etoposide

Fig.S3

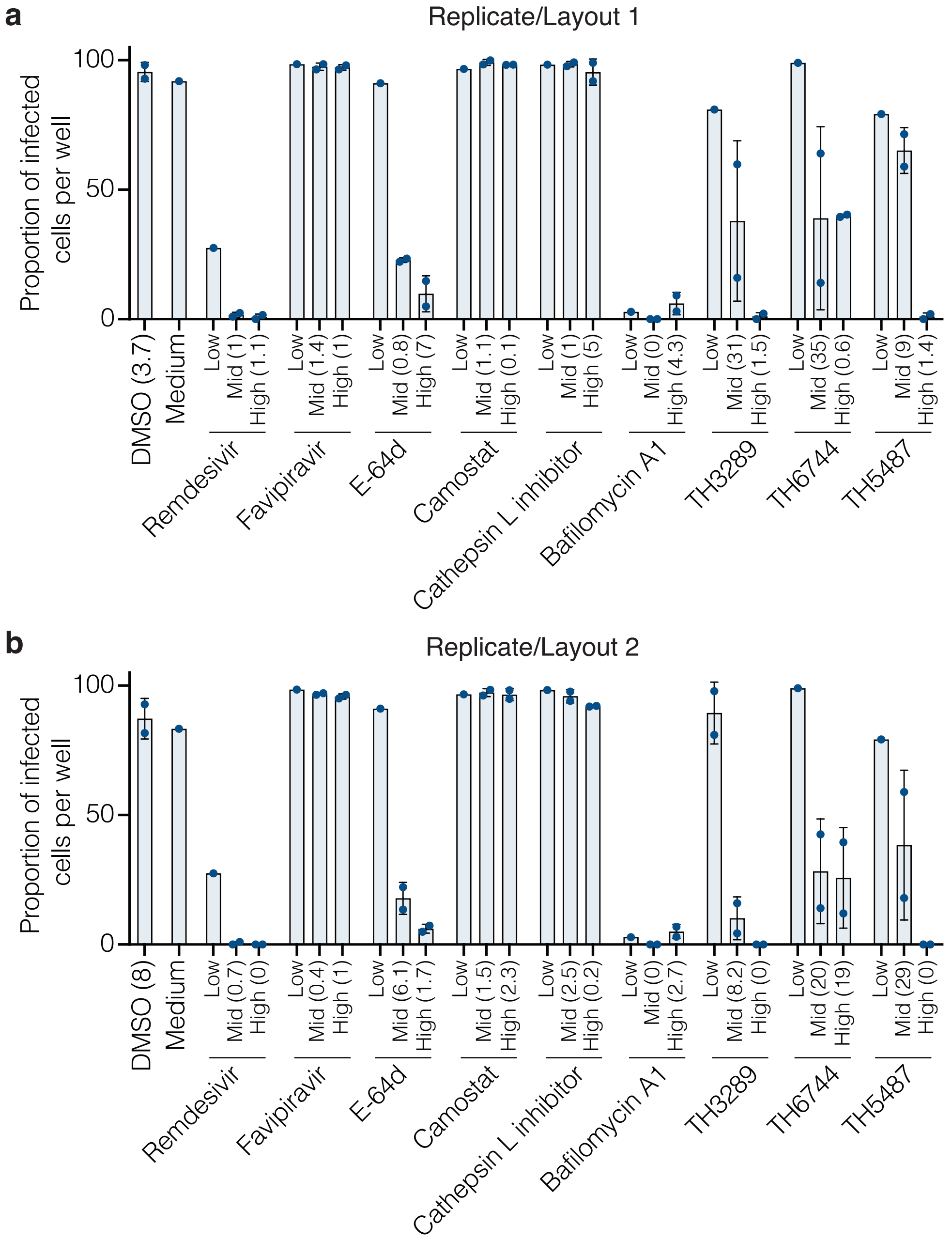
a
Replicate/Layout 1
Proportion of infected cells per well
100
50
0
DMSO (3.7)
Medium
Low
Mid (1)
High (1.1)
Remdesivir
Low
Mid (1.4)
High (1)
Favipiravir
Low
Mid (0.8)
High (7)
E-64d
Low
Mid (1.1)
High (0.1)
Camostat
Low
Mid (1)
High (5)
Cathepsin L inhibitor
Low
Mid (0)
High (4.3)
Bafilomycin A1
Low
Mid (31)
High (1.5)
TH3289
Low
Mid (35)
High (0.6)
TH6744
Low
Mid (9)
High (1.4)
TH5487
b
Replicate/Layout 2
Proportion of infected cells per well
100
50
0
DMSO (8)
Medium
Low
Mid (0.7)
High (0)
Remdesivir
Low
Mid (0.4)
High (1)
Favipiravir
Low
Mid (6.1)
High (1.7)
E-64d
Low
Mid (1.5)
High (2.3)
Camostat
Low
Mid (2.5)
High (0.2)
Cathepsin L inhibitor
Low
Mid (0)
High (2.7)
Bafilomycin A1
Low
Mid (8.2)
High (0)
TH3289
Low
Mid (20)
High (19)
TH6744
Low
Mid (29)
High (0)
TH5487

Fig.S4

a

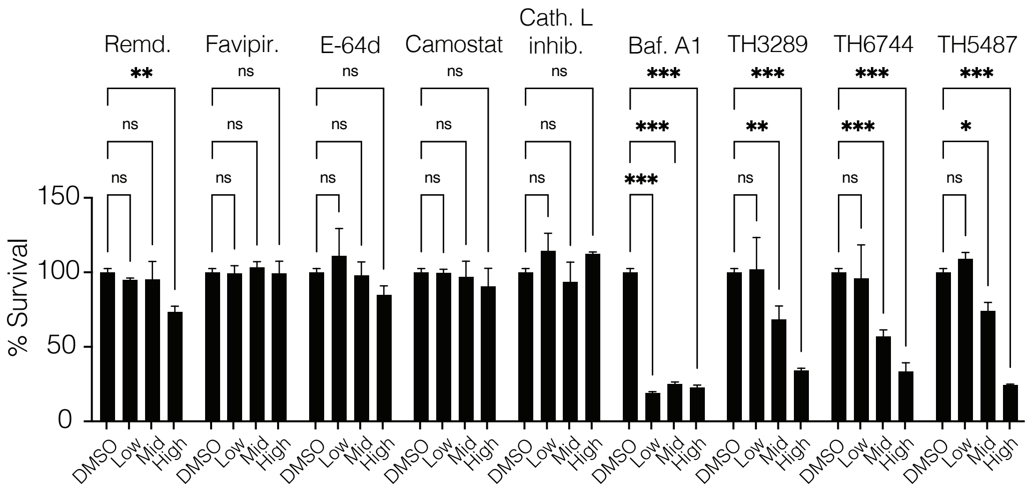
Remd.
Favipir.
E-64d
Camostat
Cath. L inhib.
Baf. A1
TH3289
TH6744
TH5487
% Survival
0
50
100
150
DMSO
Low
Mid
High
ns
*
**
***

b

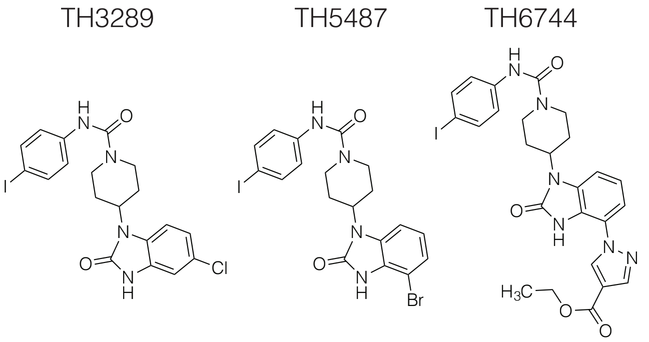
TH3289
TH5487
TH6744

c

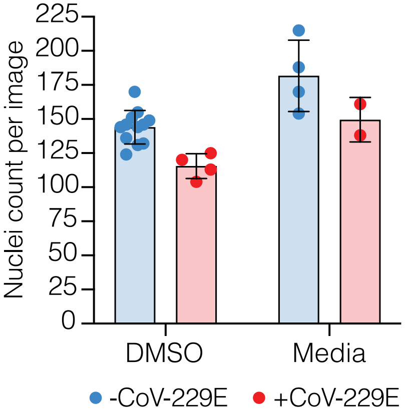
Nuclei count per image
0
25
50
75
100
125
150
175
200
225
DMSO
Media
-CoV-229E
+CoV-229E

Fig.S5

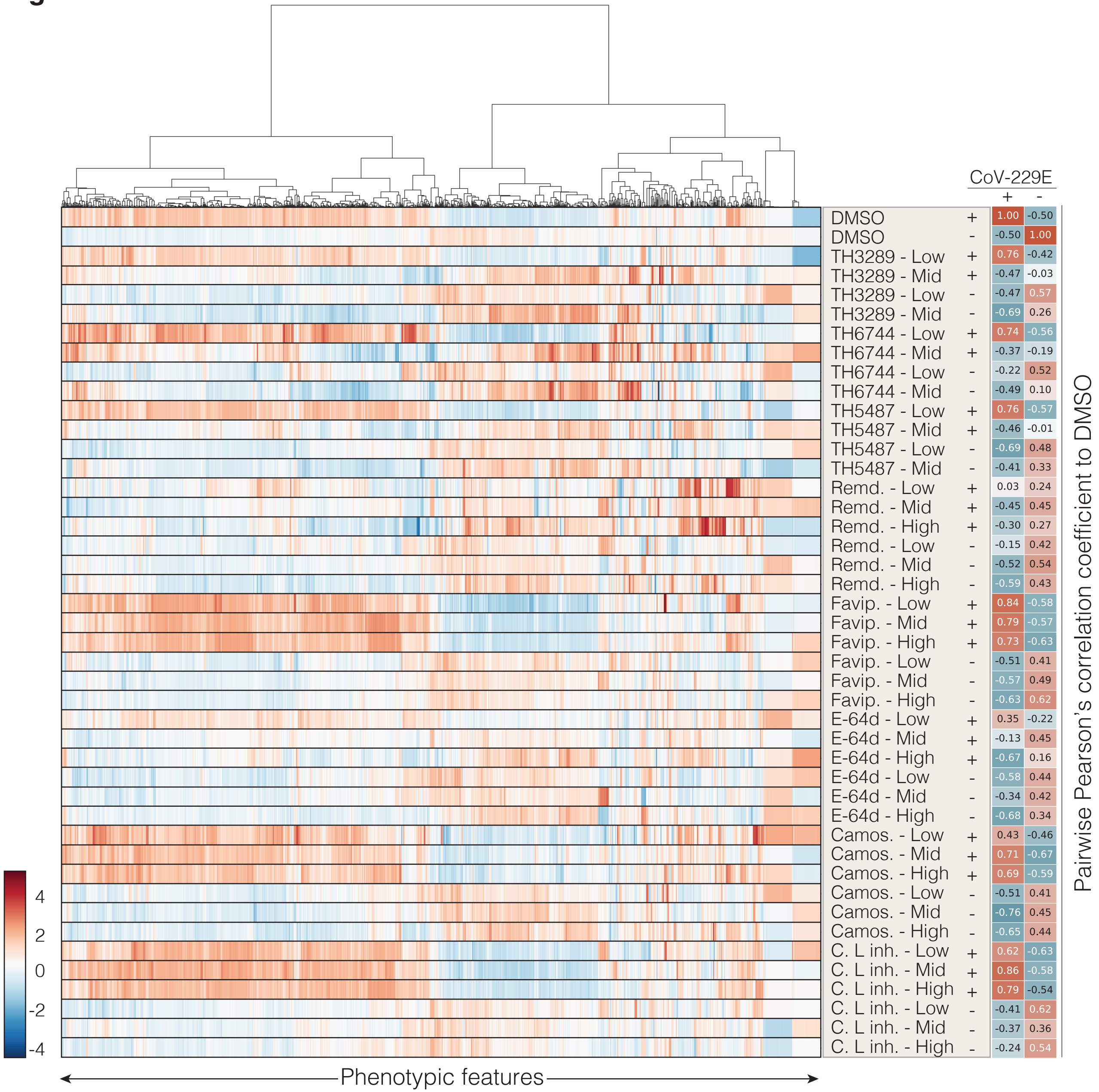
CoV-229E
+ -
DMSO + 1.00 -0.50
DMSO - -0.50 1.00
TH3289 - Low + 0.76 -0.42
TH3289 - Mid + -0.47 -0.03
TH3289 - Low - -0.47 0.57
TH3289 - Mid - -0.69 0.26
TH6744 - Low + 0.74 -0.56
TH6744 - Mid + -0.37 -0.19
TH6744 - Low - -0.22 0.52
TH6744 - Mid - -0.49 0.10
TH5487 - Low + 0.76 -0.57
TH5487 - Mid + -0.46 -0.01
TH5487 - Low - -0.69 0.48
TH5487 - Mid - -0.41 0.33
Remd. - Low + 0.03 0.24
Remd. - Mid + -0.45 0.45
Remd. - High + -0.30 0.27
Remd. - Low - -0.15 0.42
Remd. - Mid - -0.52 0.54
Remd. - High - -0.59 0.43
Favip. - Low + 0.84 -0.58
Favip. - Mid + 0.79 -0.57
Favip. - High + 0.73 -0.63
Favip. - Low - -0.51 0.41
Favip. - Mid - -0.57 0.49
Favip. - High - -0.63 0.62
E-64d - Low + 0.35 -0.22
E-64d - Mid + -0.13 0.45
E-64d - High + -0.67 0.16
E-64d - Low - -0.58 0.44
E-64d - Mid - -0.34 0.42
E-64d - High - -0.68 0.34
Camos. - Low + 0.43 -0.46
Camos. - Mid + 0.71 -0.67
Camos. - High + 0.69 -0.59
Camos. - Low - -0.51 0.41
Camos. - Mid - -0.76 0.45
Camos. - High - -0.65 0.44
C. L inh. - Low + 0.62 -0.63
C. L inh. - Mid + 0.86 -0.58
C. L inh. - High + 0.79 -0.54
C. L inh. - Low - -0.41 0.62
C. L inh. - Mid - -0.37 0.36
C. L inh. - High - -0.24 0.54
Pairwise Pearson's correlation coefficient to DMSO
4
2
0
-2
-4
Phenotypic features

Fig.S6

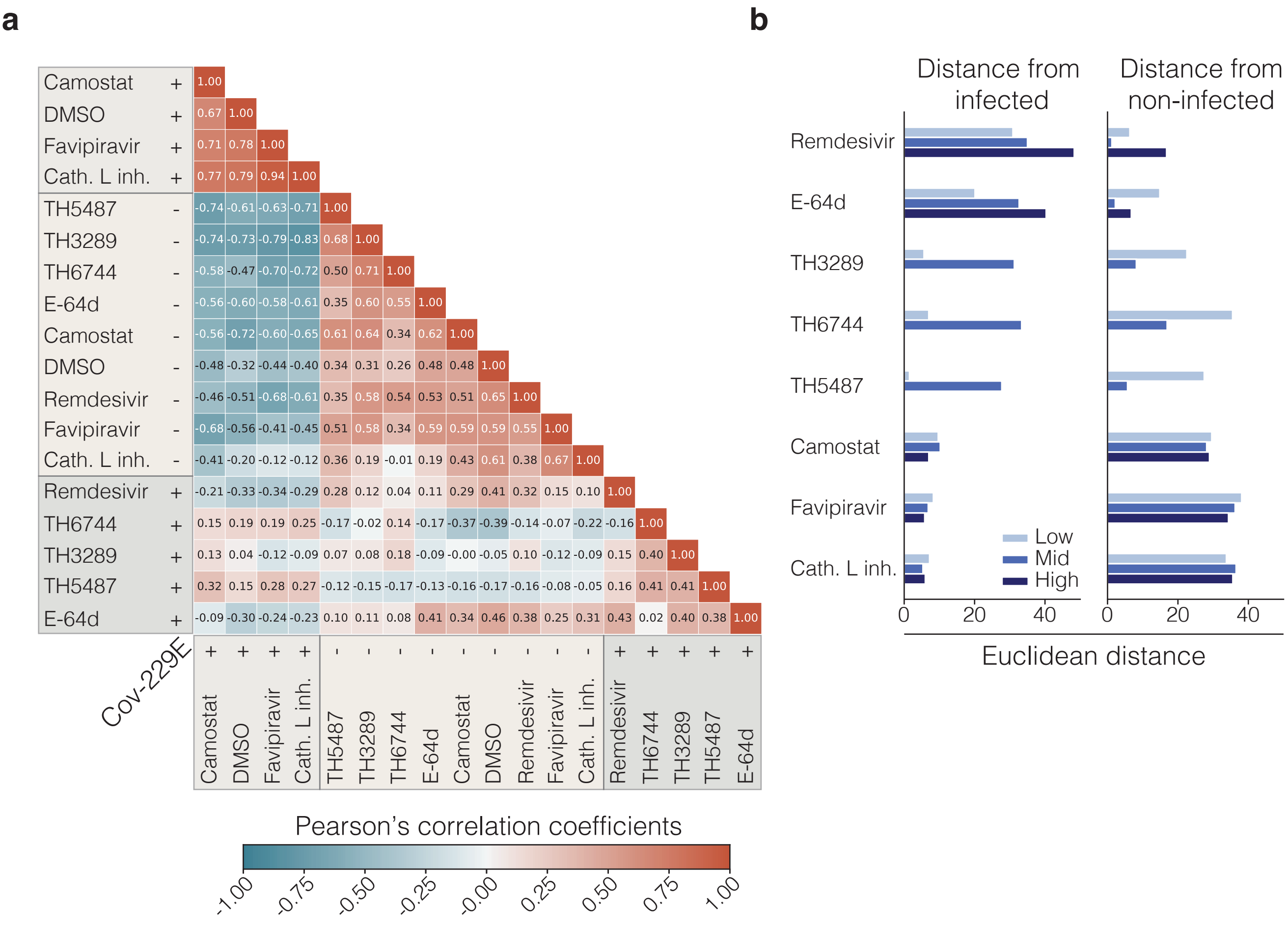
a
Camostat + 1.00
DMSO + 0.67 1.00
Favipiravir + 0.71 0.78 1.00
Cath. L inh. + 0.77 0.79 0.94 1.00
TH5487 - -0.74 -0.61 -0.63 -0.71 1.00
TH3289 - -0.74 -0.73 -0.79 -0.83 0.68 1.00
TH6744 - -0.58 -0.47 -0.70 -0.72 0.50 0.71 1.00
E-64d - -0.56 -0.60 -0.58 -0.61 0.35 0.60 0.55 1.00
Camostat - -0.56 -0.72 -0.60 -0.65 0.61 0.64 0.34 0.62 1.00
DMSO - -0.48 -0.32 -0.44 -0.40 0.34 0.31 0.26 0.48 0.48 1.00
Remdesivir - -0.46 -0.51 -0.68 -0.61 0.35 0.58 0.54 0.53 0.51 0.65 1.00
Favipiravir - -0.68 -0.56 -0.41 -0.45 0.51 0.58 0.34 0.59 0.59 0.59 0.55 1.00
Cath. L inh. - -0.41 -0.20 -0.12 -0.12 0.36 0.19 -0.01 0.19 0.43 0.61 0.38 0.67 1.00
Remdesivir + -0.21 -0.33 -0.34 -0.29 0.28 0.12 0.04 0.11 0.29 0.41 0.32 0.15 0.10 1.00
TH6744 + 0.15 0.19 0.19 0.25 -0.17 -0.02 0.14 -0.17 -0.37 -0.39 -0.14 -0.07 -0.22 -0.16 1.00
TH3289 + 0.13 0.04 -0.12 -0.09 0.07 0.08 0.18 -0.09 -0.00 -0.05 0.10 -0.12 -0.09 0.15 0.40 1.00
TH5487 + 0.32 0.15 0.28 0.27 -0.12 -0.15 -0.17 -0.13 -0.16 -0.17 -0.16 -0.08 -0.05 0.16 0.41 0.41 1.00
E-64d + -0.09 -0.30 -0.24 -0.23 0.10 0.11 0.08 0.41 0.34 0.46 0.38 0.25 0.31 0.43 0.02 0.40 0.38 1.00
Cov-229E
+ + + + - - - - - - - - - + + + + +
Camostat DMSO Favipiravir Cath. L inh. TH5487 TH3289 TH6744 E-64d Camostat DMSO Remdesivir Favipiravir Cath. L inh. Remdesivir TH6744 TH3289 TH5487 E-64d
Pearson’s correlation coefficients
-1.00 -0.75 -0.50 -0.25 -0.00 0.25 0.50 0.75 1.00
b
Distance from infected
Distance from non-infected
Remdesivir
E-64d
TH3289
TH6744
TH5487
Camostat
Favipiravir
Cath. L inh.
Low
Mid
High
0 20 40
0 20 40
Euclidean distance

Fig.S7

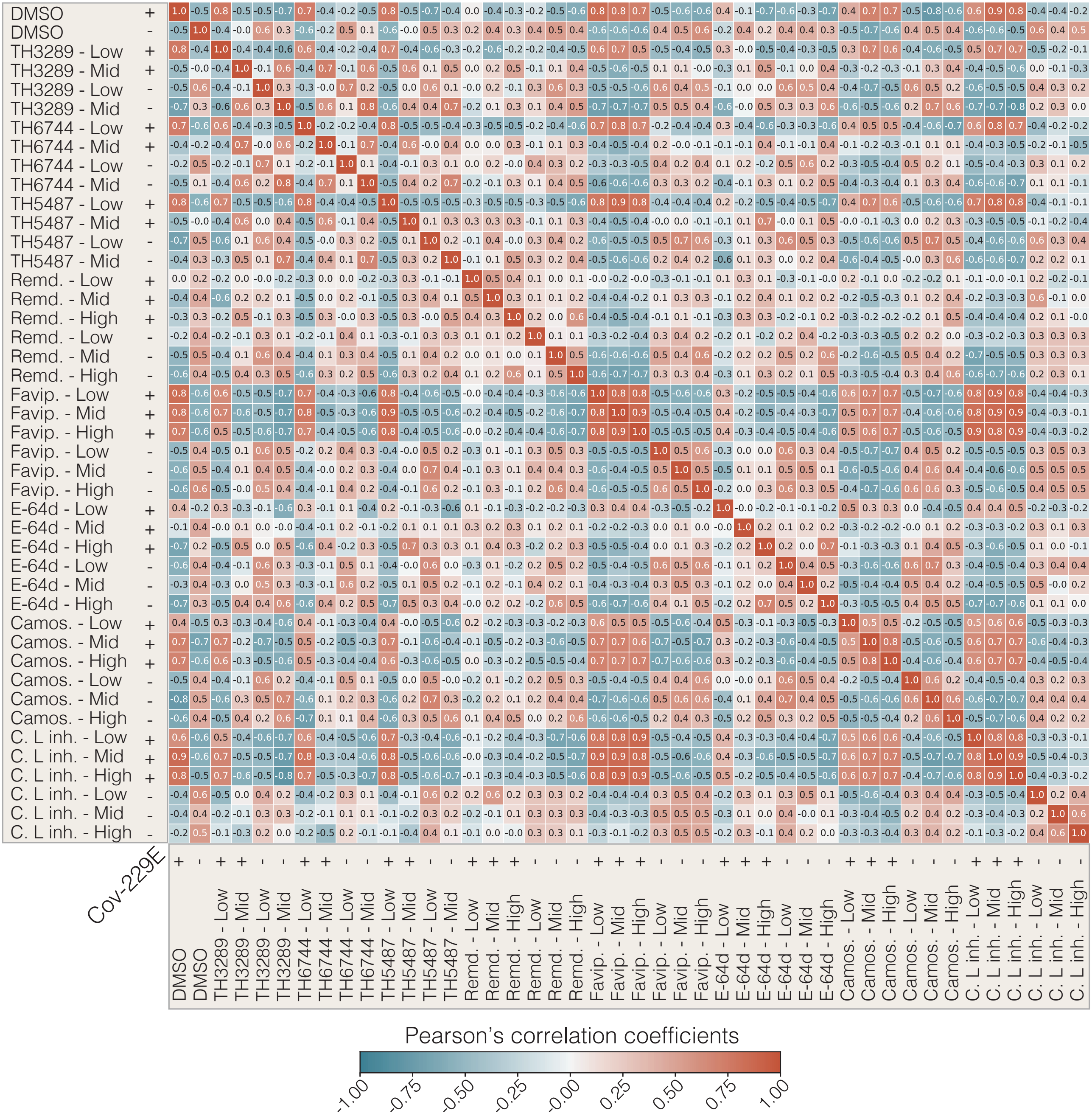
DMSO +
DMSO -
TH3289 - Low +
TH3289 - Mid +
TH3289 - Low -
TH3289 - Mid -
TH6744 - Low +
TH6744 - Mid +
TH6744 - Low -
TH6744 - Mid -
TH5487 - Low +
TH5487 - Mid +
TH5487 - Low -
TH5487 - Mid -
Remd. - Low +
Remd. - Mid +
Remd. - High +
Remd. - Low -
Remd. - Mid -
Remd. - High -
Favip. - Low +
Favip. - Mid +
Favip. - High +
Favip. - Low -
Favip. - Mid -
Favip. - High -
E-64d - Low +
E-64d - Mid +
E-64d - High +
E-64d - Low -
E-64d - Mid -
E-64d - High -
Camos. - Low +
Camos. - Mid +
Camos. - High +
Camos. - Low -
Camos. - Mid -
Camos. - High -
C. L inh. - Low +
C. L inh. - Mid +
C. L inh. - High +
C. L inh. - Low -
C. L inh. - Mid -
C. L inh. - High -
Cov-229E
Pearson's correlation coefficients
-1.00
-0.75
-0.50
-0.25
-0.00
0.25
0.50
0.75
1.00

Fig.S8

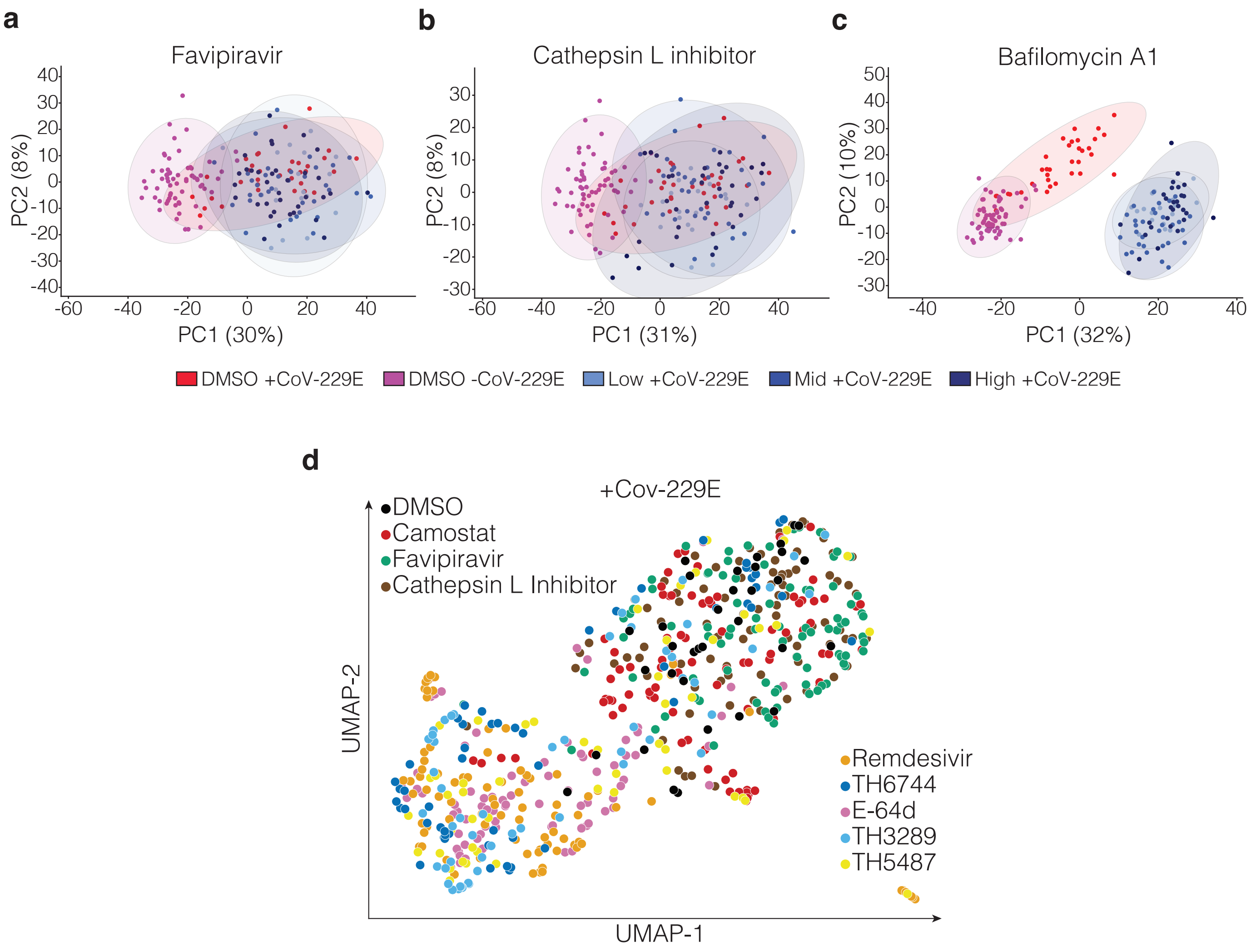
a
Favipiravir
PC2 (8%)
PC1 (30%)
b
Cathepsin L inhibitor
PC2 (8%)
PC1 (31%)
c
Bafilomycin A1
PC2 (10%)
PC1 (32%)
DMSO +CoV-229E
DMSO -CoV-229E
Low +CoV-229E
Mid +CoV-229E
High +CoV-229E
d
+Cov-229E
DMSO
Camostat
Favipiravir
Cathepsin L Inhibitor
Remdesivir
TH6744
E-64d
TH3289
TH5487
UMAP-2
UMAP-1

Fig.S9

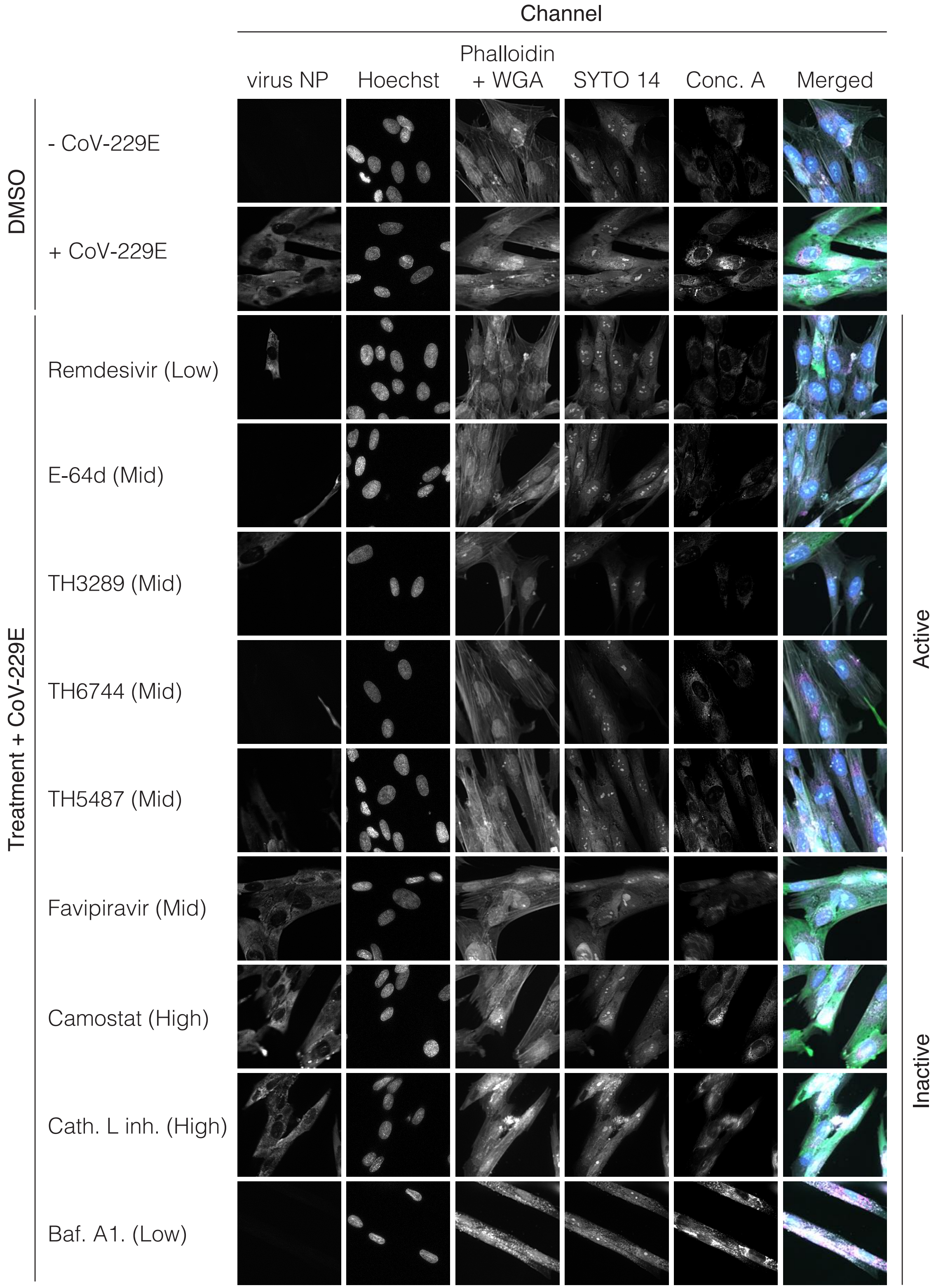

Channel
virus NP
Hoechst
Phalloidin + WGA
SYTO 14
Conc. A
Merged
DMSO
- CoV-229E
+ CoV-229E
Treatment + CoV-229E
Remdesivir (Low)
E-64d (Mid)
TH3289 (Mid)
TH6744 (Mid)
TH5487 (Mid)
Favipiravir (Mid)
Camostat (High)
Cath. L inh. (High)
Baf. A1. (Low)
Active
Inactive

Fig.S10

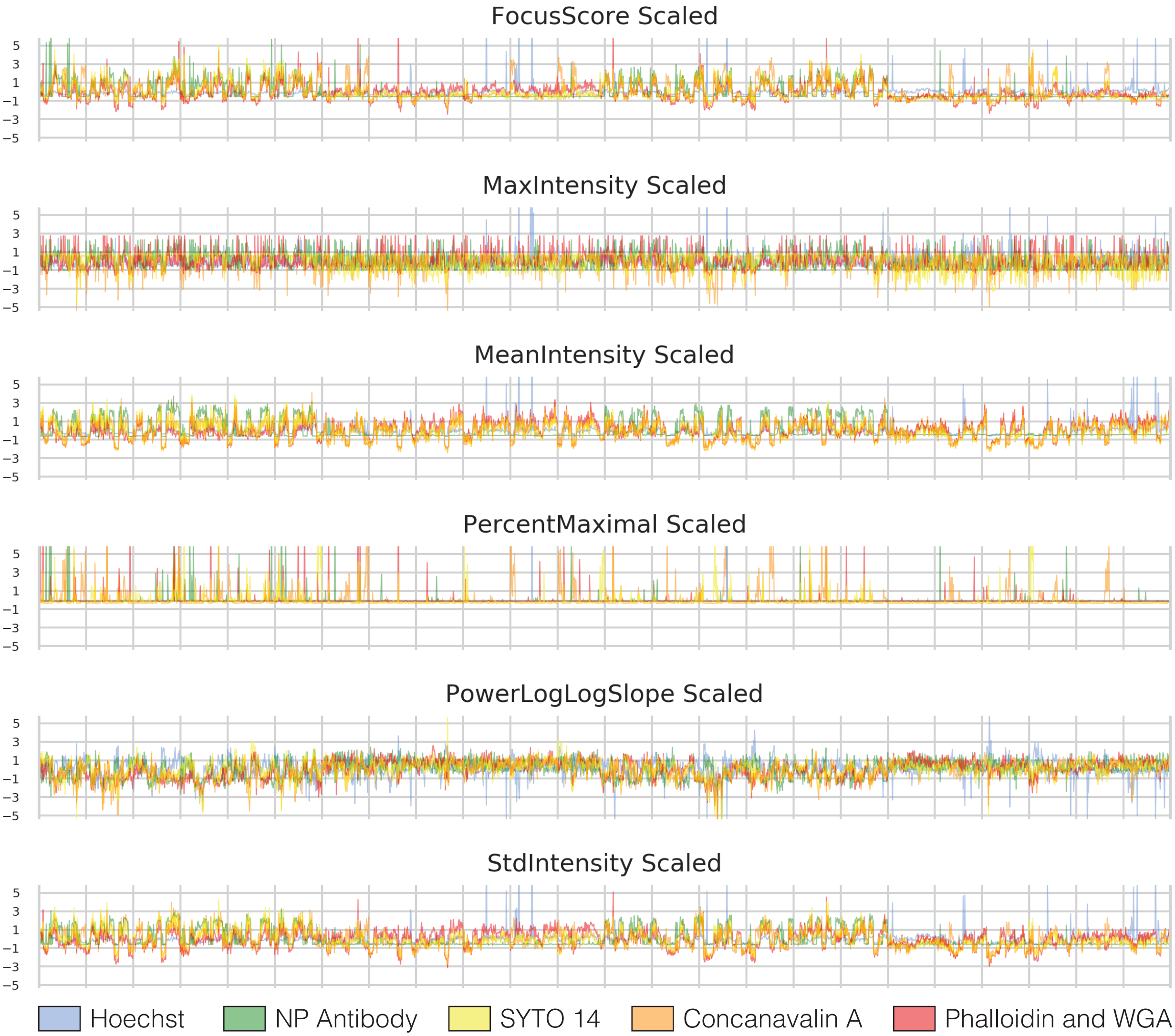
FocusScore Scaled
MaxIntensity Scaled
MeanIntensity Scaled
PercentMaximal Scaled
PowerLogLogSlope Scaled
StdIntensity Scaled
5
3
1
−1
−3
−5
Hoechst
NP Antibody
SYTO 14
Concanavalin A
Phalloidin and WGA
