# Supplementary material for: A phenomics approach for antiviral drug discovery
Source: BMC Biol. 2021 Aug 2;19:156. doi: 10.1186/s12915-021-01086-1 (PMC8325993; doi:10.1186/s12915-021-01086-1)
Supplement: Supplementary file 2 — Additional file 2: Table S1. PCA loadings of the first Principal component indicating feature importance (Fig. 1d). The top 20 positively correlated, as well as 20 most negatively correlated features including their loadings on the first principal component. [file 12915_2021_1086_MOESM2_ESM.docx]

| **Positively correlated loadings** | **PC1** |  | **Negatively correlated  loading** | **PC1** |
| --- | --- | --- | --- | --- |
| **MeanIntensity_Cell_SYTO** | 0.074255581 |  | **AreaShape_EquivalentDiameter_cell** | -0.0526401 |
| **UpperQuartileIntensity_cell_SYTO** | 0.074100557 |  | **AreaShape_Perimeter_cytoplasm** | -0.0523397 |
| **MeanIntensityEdge_cytoplasm_SYTO** | 0.072543433 |  | **AreaShape_MaxFeretDiameter_cell** | -0.0522439 |
| **UpperQuartileIntensity_cytoplasm_SYTO** | 0.072466699 |  | **AreaShape_MaxFeretDiameter_cytoplasm** | -0.0522439 |
| **MeanIntensity_cytoplasm_SYTO** | 0.072217955 |  | **AreaShape_EquivalentDiameter_cytoplasm** | -0.0519756 |
| **MedianIntensity_cell_SYTO** | 0.071093266 |  | **RadialDistribution_FracAtD_perinuclear** | -0.0516869 |
| **StdIntensityEdge_cytoplasm_SYTO** | 0.071005105 |  | **AreaShape_Perimeter_cell** | -0.0512184 |
| **MeanIntensityEdge_nuclei_SYTO** | 0.070791576 |  | **AreaShape_MajorAxisLength_cytoplasm** | -0.0501069 |
| **UpperQuartileIntensity_perinuclear_SYTO** | 0.070453333 |  | **AreaShape_MajorAxisLength_cell** | -0.0499238 |
| **MeanIntensity_perinuclear_SYTO** | 0.070204563 |  | **RadialDistribution_FracAtD_perinuclear** | -0.0492749 |
| **MeanIntensityEdge_perinuclear_SYTO** | 0.069817079 |  | **RadialDistribution_FracAtD_perinuclear** | -0.0486969 |
| **MedianIntensity_perinuclear_SYTO** | 0.068761953 |  | **RadialDistribution_FracAtD_perinuclear** | -0.0476608 |
| **MeanIntensityEdge_cytoplasm_CONC** | 0.068554908 |  | **AreaShape_CentralMoment_cell** | -0.046758 |
| **MedianIntensity_cytoplasm_SYTO** | 0.068303186 |  | **AreaShape_SpatialMoment_cell** | -0.046758 |
| **MedianIntensity_nuclei_CONC** | 0.067808394 |  | **AreaShape_Area_cell** | -0.046758 |
| **MeanIntensity_nuclei_CONC** | 0.067732508 |  | **RadialDistribution_MeanFrac_cytoplasm** | -0.0467017 |
| **UpperQuartileIntensity_cell_PHAandWGA** | 0.067605708 |  | **Granularity_1_cytoplasm** | -0.0460395 |
| **LowerQuartileIntensity_nuclei_CONC** | 0.067574965 |  | **AreaShape_CentralMoment_cytoplasm** | -0.0457216 |
| **StdIntensity_cytoplasm_SYTO** | 0.06746692 |  | **AreaShape_SpatialMoment_cytoplasm** | -0.0457216 |
| **LowerQuartileIntensity_nuclei_SYTO** | 0.067416544 |  | **AreaShape_Area_cytoplasm** | -0.0457216 |

**Table S1**
